# Annexin A1 and Dexamethasone Treatment in Hospitalized COVID-19 Patients: Impact on Disease Recovery and Evidence for an Interplay Between Proresolving Mediators

**DOI:** 10.3390/biom16040508

**Published:** 2026-03-28

**Authors:** Carolina Silva-Pereira, Marta Reina-Couto, Patrícia Pereira-Terra, Luísa Teixeira-Santos, Sandra Martins, Dora Pinho, Miguel Luz Soares, Cláudia Camila Dias, António Sarmento, Margarida Tavares, João Tiago Guimarães, José-Artur Paiva, Sónia Fraga, António Albino-Teixeira, Roberto Roncon-Albuquerque, Teresa Sousa

**Affiliations:** 1 RISE-Health, Departamento de Biomedicina–Unidade de Farmacologia e Terapêutica, Faculdade de Medicina da Universidade do Porto (FMUP), Alameda Prof. Hernâni Monteiro, 4200-319 Porto, Portugal; carolinarsp@med.up.pt (C.S.-P.); dpinho@med.up.pt (D.P.);; 2 Unidade de Investigação em Epidemiologia (EPIUnit), Instituto de Saúde Pública da Universidade do Porto, Rua das Taipas 135, 4050-600 Porto, Portugalsonia.fraga@insa.min-saude.pt (S.F.); 3 Serviço de Medicina Intensiva, Centro Hospitalar e Universitário de São João (CHUSJ), Alameda Prof. Hernâni Monteiro, 4200-319 Porto, Portugal; 4 Serviço de Farmacologia Clínica, Centro Hospitalar e Universitário de São João (CHUSJ), Alameda Prof. Hernâni Monteiro, 4200-319 Porto, Portugal; 5 Departamento de Biomedicina–Unidade de Farmacologia e Terapêutica, Faculdade de Medicina da Universidade do Porto (FMUP), Rua Dr. Plácido da Costa, S/N, Edifício Poente, Piso 3, 4200-450 Porto, Portugalats.luisa@gmail.com (L.T.-S.); 6 iNOVA4Health, NOVA Medical School (NMS), Faculdade de Ciências Médicas (FCM), Universidade NOVA de Lisboa, Campo dos Mártires da Pátria 130, 1169-056 Lisboa, Portugal; 7 Serviço de Patologia Clínica, Centro Hospitalar e Universitário de São João (CHUSJ), Alameda Prof. Hernâni Monteiro, 4200-319 Porto, Portugal; 8 Laboratório para a Investigação Integrativa e Translacional em Saúde Populacional (ITR), Rua das Taipas 135, 4050-600 Porto, Portugal; 9 Departamento de Biomedicina–Unidade de Biologia Experimental, Faculdade de Medicina da Universidade do Porto (FMUP), Rua Dr. Plácido da Costa, S/N, Edifício Poente, Piso 4, 4200-450 Porto, Portugal; 10 Instituto de Investigação e Inovação em Saúde (i3S), Universidade do Porto, Rua Alfredo Allen 208, 4200-135 Porto, Portugal; 11 RISE-Health, Departamento de Medicina da Comunidade, Informação e Decisão em Saúde (MEDCIDS), Faculdade de Medicina da Universidade do Porto (FMUP), Alameda Prof. Hernâni Monteiro, 4200-319 Porto, Portugal; camila@med.up.pt; 12 Departamento de Recursos Comuns–Unidade de Gestão de Conhecimento, Faculdade de Medicina da Universidade do Porto (FMUP), Alameda Prof. Hernâni Monteiro, 4200-319 Porto, Portugal; 13 Serviço de Doenças Infecciosas, Centro Hospitalar e Universitário de São João (CHUSJ), Alameda Prof. Hernâni Monteiro, 4200-319 Porto, Portugal; 14 RISE-Health, Departamento de Medicina, Faculdade de Medicina da Universidade do Porto (FMUP), Alameda Prof. Hernâni Monteiro, 4200-319 Porto, Portugal; 15 Departamento de Biomedicina—Unidade de Bioquímica, Faculdade de Medicina da Universidade do Porto (FMUP), Alameda Prof. Hernâni Monteiro, 4200-319 Porto, Portugal; 16 Departamento de Medicina, Faculdade de Medicina da Universidade do Porto (FMUP), Alameda Prof. Hernâni Monteiro, 4200-319 Porto, Portugal; 17 Departamento de Saúde Ambiental, Instituto Nacional de Saúde Doutor Ricardo Jorge, Rua Alexandre Herculano 321, 4000-055 Porto, Portugal; 18 RISE-Health, Departamento de Cirurgia e Fisiologia, Faculdade de Medicina da Universidade do Porto (FMUP), Alameda Prof. Hernâni Monteiro, 4200-319 Porto, Portugal

**Keywords:** annexin A1, FPR2, dexamethasone, resolvin E1, critical COVID-19

## Abstract

Annexin A1 (ANXA1) is a proresolving protein regulated by glucocorticoids, the standard care for severe and critical COVID-19 patients. As part of a larger project including hospitalized COVID-19 patients, this study aimed at evaluating ANXA1 and its FPR2 receptor in these patients, focusing on longitudinal profiles and comparison across disease severities and outcomes, and exploring their correlations with inflammation, endotheliitis and other proresolving mediators. Blood was collected in “severe” (*n* = 27), “critical” (*n* = 17) and “critical on veno-venous extracorporeal membrane oxygenation” (*n* = 17) COVID-19 patients at admission, days 3–4, 5–8, and weekly thereafter, and in controls (*n* = 23) at a single time point. We quantified ANXA1, resolvin D1, resolvin E1 (RvE1) and endocan by ELISA, cytokines and other endothelial markers by multiplex immunoassays, and FPR2 and Chemerin_1_ receptors by RT-qPCR. Most patients underwent a 10-day dexamethasone regimen. Admission ANXA1 and *FPR2* were significantly higher in all patient groups. Throughout hospitalization, ANXA1 increased mainly in “severe” patients and survivors, becoming higher at weeks 3 and 4 in survivors versus non-survivors. Variable cumulative dexamethasone doses did not differentially affect ANXA1 or *FPR2*. ANXA1 was associated with higher RvE1 during the dexamethasone effect period. Exploratory analyses showed that ANXA1 inversely correlated with RvE1 receptor and endotheliitis, whereas both ANXA1 and *FPR2* positively correlated with inflammation. In conclusion, ANXA1 may be involved in COVID-19 recovery processes, and its interplay with RvE1 may ameliorate hyperinflammation.

## 1. Introduction

The COVID-19 pandemic led to a unified effort by the scientific community to better understand the human infection caused by the severe acute respiratory syndrome coronavirus 2 (SARS-CoV-2), which ranges from being asymptomatic to causing severe clinical conditions and death [1]. Hospitalization is required for severe and critical cases, with critical COVID-19 patients needing life-sustaining therapies, such as mechanical ventilation or even veno-venous extracorporeal membrane oxygenation (VV-ECMO) [2]. Current knowledge on the pathophysiology of COVID-19 highlights a complex interplay of dysregulated inflammatory responses, endothelial dysfunction and hypercoagulation, contributing to disease severity and multi-organ complications [3,4]. Inflammatory disturbances may involve both excessive production of proinflammatory factors, such as cytokines, and an impaired resolution process [5,6]. The latter, known as resolution of inflammation, normally encompasses the stimulation of neutrophil apoptosis, clearance of apoptotic granulocytes by monocytes and macrophages in a non-inflammatory manner, and finally, the removal of leukocytes and debris from inflamed sites, enabling the restoration of tissue homeostasis. This is achieved through the active coordination of proresolving mediators, including lipids, specialized proresolving mediators (SPMs; e.g., lipoxins; resolvins; protectins; maresins), proteins and peptides (e.g., annexin A1, ANXA1; galectin-1), and gases (e.g., H_2_S; CO) [7,8]. In fact, both proinflammatory and proresolving mediators have been found to be altered in COVID-19 patients, particularly in those with critical illness [9,10,11,12]. Therefore, therapeutic strategies targeting inflammatory pathways, like glucocorticoids, IL-6 receptor blockers, Janus kinase inhibitors, or, more recently, TNF-α inhibitors, rather than antiviral drugs, are preferentially recommended as disease severity increases [13,14]. Among these, glucocorticoids have gained significant attention for their positive outcomes in clinical trials and have been recommended as standard care for severe or critical COVID-19 patients [13,14].

ANXA1, a member of the superfamily of annexins, was first identified as a glucocorticoid-induced protein capable of inhibiting phospholipase A_2_, and thereby reducing eicosanoid production [15], which prompted its study as an anti-inflammatory agent [16,17]. ANXA1 is expressed in the cytoplasm of peripheral blood cells, mainly in neutrophils and monocytes, and is also found in epithelial cells (e.g., in the lung, gut and kidney) and in endothelial cells [18,19]. Upon cell activation or various stimuli, ANXA1 is secreted and can be detected in biological fluids, such as plasma, serum and alveolar lavage fluids [20,21]. Glucocorticoids are the primary stimulus for ANXA1, not only inducing its de novo synthesis but also promoting its translocation to the cell surface [22]. Once externalized, ANXA1 binds to its target, the G protein-coupled receptor FPR2, activating signaling pathways that modulate the inflammatory response [20,23]. Interestingly, *FPR2* expression is also induced by glucocorticoids [24], underscoring the pivotal role of the ANXA1-FPR2 pathway in mediating their anti-inflammatory and proresolving effects [22]. FPR2 is also activated by the lipid mediators resolvin D1 (RvD1) and lipoxin A_4_ (LXA_4_) [25], and evidence has suggested the existence of crosstalk among its ligands [26,27,28,29]. Additionally, resolvin E1 (RvE1), which activates chemerin receptor 1 (Chemerin_1_) [30], has been reported to stimulate LXA_4_ production [31], further supporting the existence of an interconnected network between proresolving mediators.

ANXA1 orchestrates key events in the transition from acute inflammation to its resolution [32] and these effects underlie the protective role that ANXA1 has demonstrated in various infectious diseases, including bacterial, viral, and parasitic [33]. Specifically in respiratory infections, ANXA1-deficient mice with tuberculosis or pneumococcal pneumonia exhibited increased bacterial proliferation and lung damage [34,35,36] and these detrimental effects were reversed by treatment with the ANXA1 mimetic peptide Ac2-26, acting through FPR2 [36]. Extending these findings to coronavirus infections, Resende et al. recently also showed exacerbated pathology in ANXA1-deficient mice infected with betacoronavirus MHV-3, and Ac2-26 treatment also reduced lung damage and lethality in SARS-CoV-2-infected mice [37]. Beyond infectious diseases, treatment with ANXA1 or Ac2-26 was also associated with reduced inflammation and lung injury in models of non-infectious pulmonary disease [38,39,40]. In humans, ANXA1 expression or circulating concentrations have been reported as either elevated [41,42] or reduced [43,44,45] in patients with various infections compared to healthy controls, with both profiles linked to disease severity [41,43,46,47,48]. Similarly, the few studies investigating ANXA1 in COVID-19 patients have yielded conflicting results when analyzing its concentration profiles in patients vs. healthy controls, as well as its association with disease severity [49,50,51,52]. This limited and contradictory evidence on ANXA1 in COVID-19, combined with the notable efficacy of glucocorticoids in critically ill patients, highlights the need for further investigation of the ANXA1-FPR2 pathway in COVID-19.

Integrated in a broader research project, this study’s primary objective was to evaluate ANXA1 concentrations and *FPR2* expression in hospitalized COVID-19 patients (the majority of whom were treated with dexamethasone), comparing their longitudinal profiles during hospitalization across different disease severities and between survivors and non-survivors. Additionally, as a secondary objective, we conducted exploratory analyses to investigate correlations between the ANXA1-FPR2 axis and inflammatory status and endothelial dysfunction, as well as other proresolving mediators or proresolving receptors, which remain poorly explored.

## 2. Materials and Methods

### 2.1. Study Design and Population

This study is part of a larger research project (RESEARCH 4 COVID-19 grant, project 519-reference number 613690173, “Unresolved inflammation and endothelitis in severe COVID-19 patients: identification of risk stratification biomarkers and therapeutic targets”, FCT—Fundação para a Ciência e a Tecnologia) involving patients from the ward of the Service of Infectious Diseases, as well as from the intensive care units (ICU) of both the Service of Intensive Care Medicine and the Service of Infectious Diseases of a tertiary hospital (Centro Hospitalar Universitário São João, CHUSJ) [12,53,54]. A total of sixty-one patients (*n* = 61) of both sexes hospitalized with hypoxemic respiratory failure who were symptomatic for >1 day were consecutively enrolled between September 2020 and February 2021, following a laboratory-confirmed diagnosis of SARS-CoV-2 infection by a positive RT-PCR result from a nasopharyngeal swab. Most patients were recruited within 72 h of a positive RT-PCR test. Exclusion criteria included age under 18, pregnancy or lactation, and a history of vasculitis or connective tissue disease. Admission to the ward or ICU and decision and time for intubation, mechanical ventilation and/or VV-ECMO were based on clinical judgment according to “lege artis”. Patients were divided into two groups based on COVID-19 disease severity [2]: patients with severe COVID-19 (*n* = 27) admitted to the ward and patients with critical COVID-19 (*n* = 34) admitted to the ICU. The definitions of severe and critical disease were based on the World Health Organization’s guidelines published during the recruitment period, and these are still in line with the most recent guidelines from the Infectious Diseases Society of America, where severe and critical patients are categorized according to ventilatory support [13]. The patients with critical COVID-19 were further subdivided into two groups, depending on the need for VV-ECMO support: group of critically ill COVID-19 patients without VV-ECMO support (critical COVID-19, *n* = 17) and group of critically ill COVID-19 patients on VV-ECMO (critical COVID-19 on VV-ECMO, *n* = 17). The prospective design of our sampling allowed us to capture a heterogeneous population of ward and ICU patients. Controls (*n* = 23) were recruited prior to the COVID-19 pandemic among healthy blood donor volunteers from the Service of Immunohemotherapy of CHUSJ. Ethical approval and informed consent procedures for this cohort have been previously described [54], and all participants provided informed consent.

### 2.2. Clinical Data and Sample Collection

Throughout their stay in the ward or ICU, all patients were followed by the project medical team, who recorded relevant clinical and demographic parameters. All data were anonymously coded and added to the project database to ensure confidentiality. The Acute Physiology and Chronic Health Evaluation II (APACHE II) and Simplified Acute Physiology Score II (SAPS II) scoring systems were calculated to estimate illness severity at ICU admission. Total lengths of hospital and ICU stay were also assessed. These calculations included hospitalizations at CHUSJ as well as at other hospitals where some patients had been admitted before transfer to CHUSJ or to which they were transferred afterwards. Total length of hospital stay was defined as the entire continuous hospitalization period, including both ward and ICU stays, whereas length of ICU stay corresponded only to the total time spent in the ICU. As a tertiary hospital, CHUSJ was frequently requested for specialized care during the pandemic, requiring strict capacity management. Therefore, patients were often transferred to hospitals closer to their residences once tertiary-level care was no longer necessary. In addition, the critical COVID-19 on VV-ECMO group included 11 patients who had been admitted to ICUs of other hospitals before being transferred to the ICU of CHUSJ for VV-ECMO cannulation, as CHUSJ is a national reference center for ECMO in Portugal. Intrahospital mortality was reported by the medical team, confirming that all non-survivors died during hospitalization due to COVID-19 or related complications.

For all patients, blood samples were collected at different time points throughout their hospital stay at CHUSJ, whenever possible: up to 48 h (days 1–2; admission), on days 3–4, on days 5–8 after admission and weekly thereafter until hospital discharge or a negative result in RT-PCR COVID-19 test. All samples from critical COVID-19 patients on VV-ECMO were collected after VV-ECMO initiation. Samples (blood) from controls were collected at a single time point. All samples were processed within 1–2 h of collection and stored at −80 °C until assayed. To obtain serum, blood samples were collected in tubes containing a clot activator and a separating gel, then allowed to coagulate for 2 h at room temperature and centrifuged at 1000× *g* for 20 min at 4 °C, after which the serum was aliquoted and stored until analysis. Blood samples were also collected in EDTA tubes, and peripheral blood mononuclear cells (PBMCs) and polymorphonuclear cells (PMNs) were isolated using Polymorphprep^TM^ (Axis-Shield, Oslo, Norway) according to the manufacturer’s instructions. Briefly, whole blood was carefully layered onto Polymorphprep^TM^ and centrifuged at 450× *g* for 30 min at room temperature without brake to separate leukocyte populations. The PBMC and PMN layers were recovered, washed twice in saline solution (400× *g*, 10 min, 18–20 °C) and resuspended in Trizol for storage. Due to insufficient PMN yield and RNA quality, subsequent mRNA quantification was performed exclusively on PBMCs.

### 2.3. Evaluation of s-ANXA1, Resolvins and Relative Quantification of FPR2 and Chemerin Receptor 1

Quantification of serum ANXA1 (s-ANXA1) was performed by an enzyme-linked immunosorbent assay (ELISA) using the commercial kit “Annexin A1 (human) ELISA Kit, Item No. 501550” from Cayman Chemical Company, Ann Arbor, MI, USA.

Serum resolvin D1 (s-RvD1) and serum resolvin E1 (s-RvE1) were measured by ELISA using the commercial kits “Human Resolvin D1 (RvD1) ELISA kit, Cat. No: MBS756429” and “Human Resolvin E1 (RvE1) ELISA Kit, Cat. No: MBS025958”, respectively, from MyBioSource, Inc., San Diego, CA, USA. Resolvin quantification was performed according to the manufacturer’s instructions, and detailed information regarding assay procedures and specificity has been previously reported [54].

*FPR2* and *CMKLR1* (Chemerin_1_ gene) were quantified by Real-Time quantitative Polymerase Chain Reaction (RT-qPCR). The NZY Total RNA Isolation kit (NZYTECH, Lisbon, Portugal) was applied according to the manufacturer’s instructions to extract total RNA from PBMCs. Then, RNA was quantified using Nanodrop 2000 (ThermoFisher Scientific, Waltham, MA, USA), and its integrity was assessed using the Agilent 2100 Bioanalyzer (Agilent Technologies, Inc., Santa Clara, CA, USA). The reverse transcription of 500 ng of total RNA was performed with random primers using the NZY First-STRAND cDNA Synthesis Kit (NZYTECH, Lisbon, Portugal), and the resulting cDNA was diluted 1:20, aliquoted and stored at 4 °C for subsequent use. The expression levels of the selected genes were measured by RT-qPCR using the StepOnePlus Real-Time PCR System (Applied Biosystems, ThermoFisher Scientific, Waltham, MA, USA). Reaction conditions, primer concentrations, and quantification procedures have been previously described [54]. Relative gene expression was calculated by normalizing target gene expression to the expression of *GAPDH* within each sample, and results are expressed as target gene/*GAPDH* relative mRNA expression (arbitrary units). The following primer sequences were used for detecting transcripts: *GAPDH*, F: 5′-CCATCACCATCTTCCAGGAG-3′, R: 5′-GCATGGACTGTGGTCATGAG-3′; *FPR2*, F: 5′-AGCCCCAACTAATGACACGG-3′, R: 5′-TGACCCCATCCTCACATTGC-3′; *CMKLR1*, F: 5′-CATCATCAGCTCTGACCGCT-3′, R: 5′-TGTCCCGGAAGACGAGAGAT-3′.

### 2.4. Quantification of Routine Laboratory Markers, Cytokines and Endothelial Activation Markers

All the routine laboratory analyses were performed at the Service of Clinical Pathology of CHUSJ. Arterial blood gas analysis was used to quantify lactate, partial pressure of oxygen (PaO_2_), and partial pressure of carbon dioxide (PaCO_2_). For the calculation of the PaO_2_/FiO_2_ ratio, the fraction of inspired oxygen (FiO_2_) was obtained from the oxygen administration device and oxygen dose information in the medical records. Serum C-reactive protein (s-CRP) was quantified by an immunoturbidimetric assay using a Beckman Coulter^®^ AU5800 automated clinical chemistry analyzer (Beckman-Coulter, Hamburg, Germany). Differential leukocyte count (leukocytes, neutrophils, monocytes and lymphocytes) was analyzed by flow-cytometry in an automated hematology analysis system (Sysmex 5000; Emílio de Azevedo Campos, Porto, Portugal). As we lacked permission to access hospital laboratory reports for the control group, their routine clinical biomarkers were not included in this study.

Serum cytokines (s-TNF-α, s-IL-1β, s-IL-6 and s-IL-10) and endothelial activation markers (serum vascular cell adhesion molecule 1, s-VCAM-1, and serum E-selectin, s-E-selectin) were evaluated by multiplex immunoassays using a Luminex 200 analyzer (Luminex Corporation, Austin, TX, USA) according to the protocols of MILLIPLEX^®^ MAP Human High Sensitivity T Cell Magnetic Bead Panel (Millipore Corporation, Billerica, MA, USA) and Luminex Human Magnetic Assay (R&D Systems, Inc., Minneapolis, MN, USA), respectively. Raw data analysis (mean fluorescence intensity) was performed using ISTM 2.3 software (Luminex Corporation, Austin, TX, USA). Serum endocan (s-Endocan) was measured by ELISA using the commercial kit “Just Do It ELISA Kit H1” (JDIEK H1 assay, Lunginnov s.a.s, Lille, France).

### 2.5. Statistical Analysis

Univariate statistical analysis was conducted using the GraphPad Prism 10 software (La Jolla, CA, USA). Results are expressed as mean ± standard error of the mean (SEM) or as median (25th percentile; 75th percentile) for data with normal or non-normal distribution, respectively, or as a percentage. They are graphically represented as Box-and-Whiskers plots. For comparisons between two groups, results were analyzed by unpaired Student’s *t*-test for parametric data or the Mann–Whitney U-test for nonparametric data. For comparisons involving three or more groups, parametric data were analyzed using one-way ANOVA followed by Tukey’s multiple comparisons test, whereas nonparametric data were analyzed using the Kruskal–Wallis test followed by Dunn’s post hoc test. Categorical variables were analyzed by the chi-square test. Each biomarker’s longitudinal evolution was analyzed by mixed-effects analysis with the Geisser–Greenhouse correction, followed by Tukey’s multiple comparison test for parametric data or the Wilcoxon matched pairs signed rank test for nonparametric data. Statistical analysis was restricted to data collected up to week 4 of hospitalization. Spearman’s correlation analyses were used to estimate correlations between sets of nonparametric data among all patients during hospitalization, and *p*-values were adjusted for multiple testing using the Benjamini–Hochberg false discovery rate correction. The longitudinal and correlation analyses were conducted as exploratory and hypothesis-generating approaches for future studies. *p*-values < 0.050 were considered significant.

Repeated measures multivariate analyses adjusted for age and sex, using the IBM SPSS Statistics 27 software (IBM Corporation, New York, NY, USA), were conducted to examine associations with s-ANXA1 (dependent variable) during hospitalization among all patients. To address our primary objectives, we assessed the relationships between s-ANXA1 and the COVID-19 patient group and intrahospital mortality. For the secondary objectives, we examined the association between s-ANXA1 and s-RvE1.

All patients were assessed by the same medical team involved in the project to minimize possible bias in clinical evaluation. To ensure comparability of biomarker measurements, samples from controls and patient groups were balanced across assay plates. Some biomarker values were unavailable due to insufficient sample volume or lack of reagents. Supplementary Table S1 shows the final sample size per group for each biomarker evaluated at admission. The number of patients and collected samples decreased over time due to death, withdrawal of consent or hospital discharge, as well as logistical constraints affecting sample collection at specific timepoints (Supplementary Figure S1). Missing data were not imputed to avoid potential bias.

As stated above, this study was conducted within the framework of a broader research project funded by FCT aimed at identifying novel biomarkers and potential therapeutic targets in COVID-19, in which multiple parameters were evaluated within the same cohort. Therefore, distinct biological hypotheses within a unified research framework were addressed in different publications, where demographic, clinical and routine laboratory data overlap. Moreover, some inflammatory, endothelial and proresolving parameters have been used in these publications to enable exploratory correlation analyses with the primary biomarkers under investigation [12,53,54]. The present study presents for the first time data on ANXA1 concentrations and *FPR2* expression throughout hospitalization across COVID-19 severity groups and outcomes, as well as correlation analyses between ANXA1/*FPR2* and inflammatory/endothelial/proresolving parameters, and multivariate analyses of ANXA1. Of the ANXA1-FPR2 pathway, only the *FPR2* expression in controls and all patients at admission was previously reported [54].

Sample size was defined according to the primary objectives of our FCT-funded RESEARCH 4 COVID-19 project, which consisted of characterizing the resolution of inflammation and endotheliitis. Based on preliminary evaluations of specialized proresolving mediators in healthy controls and patients with severe and critical disease, we deemed a standardized effect size of ca. 0.9 to be clinically relevant and, using power analysis, determined a sample size of 21 subjects per group to obtain an 80% power at a 5% significance level. However, during the recruitment process, a high number of critically ill patients were included, and VV-ECMO patients constituted ca. half of them (CHUSJ is a reference center for ECMO, and many critically ill patients were transferred for VV-ECMO cannulation). Furthermore, preliminary evaluations revealed marked heterogeneity in values between critically ill patients with and without VV-ECMO support. Therefore, we decided to divide the group of patients with critical COVID-19 into two groups: critically ill (without VV-ECMO) and critically ill on VV-ECMO. Overall, a total number of 84 subjects (i.e., 4 times the initially estimated 21 subjects) was therefore included in the study, albeit with only 17 patients per group in the two critically ill groups. Reporting of the study conforms to the STROBE statement, along with references to STROBE and the broader EQUATOR guidelines [55].

## 3. Results

### 3.1. Population Demographic and Clinical Characterization

Demographic and clinical characteristics of the subjects included in the study are presented in Table 1.

There were significant age differences between groups, with severe COVID-19 patients being older than controls (*p* < 0.010) and critically ill COVID-19 on VV-ECMO patients being younger than severe and critically ill COVID-19 patients (*p* < 0.001 and *p* < 0.050, respectively). We did not find significant differences in the proportion of men and women between groups, but men were predominant in all groups. Arterial hypertension was the most prevalent comorbidity in severe and critically ill COVID-19 patients, while obesity predominated in critical COVID-19 on VV-ECMO patients. Nevertheless, no significant differences were found in comorbidities between patient groups. When comparing both groups of critically ill patients, there were no significant differences in APACHE II and SAPS II prognostic scores.

At admission, almost all patients initiated treatment with dexamethasone and very few with remdesivir, with no significant differences between groups. Patients received dexamethasone for 10 consecutive days according to the guidelines in force during the study period [56]. All blood collections occurred after treatment initiation, but the timing varied among patients, resulting in different cumulative doses of dexamethasone at each time point (Supplementary Figure S2). Most treated patients (74%) initiated dexamethasone within 3 days before the first blood collection, thus completing treatment by the beginning of the second week of hospitalization. However, critical COVID-19 on VV-ECMO patients who were previously hospitalized in the ICUs of other hospitals initiated corticotherapy prior to transfer to the ICU of CHUSJ, thereby completing treatment earlier during their CHUSJ stay, with five of them having already completed treatment before the first blood sample was obtained (Supplementary Figure S2). In weeks 3 and 4 of hospitalization, no patients were under the effect of dexamethasone, according to its biological half-life (Supplementary Figure S2). Additionally, both groups of critically ill COVID-19 patients (with or without VV-ECMO) presented a tendentially higher proportion of patients receiving antibiotics compared to the severe COVID-19 group (*p* = 0.051).

Admission lactate values did not differ between patient groups. As expected, both groups of critically ill patients presented a significantly lower PaO_2_/FiO_2_ ratio compared to those with severe COVID-19 (*p* < 0.001), as well as significantly higher PaCO_2_, especially in those in the VV-ECMO group. Hence, there was a higher need for mechanical ventilation, non-invasive ventilation and high-flow cannula oxygen in all critical COVID-19 patients (with or without VV-ECMO) when compared with severe COVID-19 patients (*p* < 0.001, *p* < 0.001 and *p* = 0.020, respectively).

Both groups of critically ill patients had a longer length of stay in the ICU than severe patients (*p* < 0.001), because among the 27 severe COVID-19 patients, only five needed a temporary upgrade of care to the ICU in the first week of hospitalization [median length of ICU stay: 11 (3; 36) days]. At ICU admission, those five patients had mean APACHE II and SAPS II scores of 11 ± 1 and 28 ± 4, respectively. Additionally, between groups of critically ill COVID-19 patients, those on VV-ECMO had a longer length of ICU stay, although this did not reach statistical significance. Both groups of critically ill patients also had a longer total length of hospital stay when compared to severe COVID-19 patients (*p* < 0.001 and *p* < 0.010, respectively). Regarding intrahospital mortality, no significant differences were observed between COVID-19 patient groups.

### 3.2. ANXA1 at Admission and During Hospitalization

**Figure 1:**
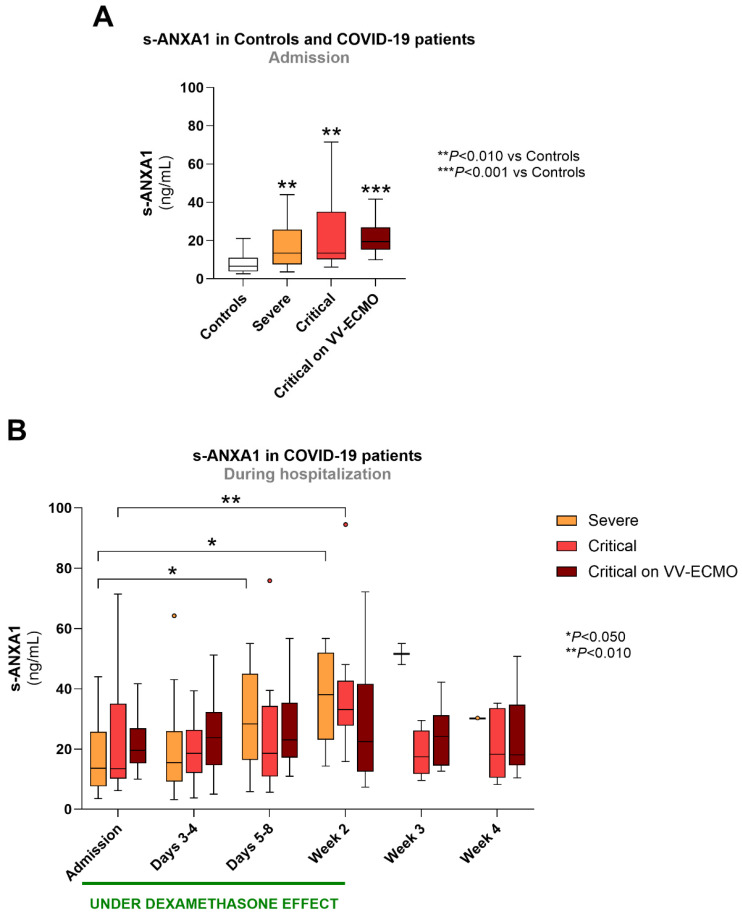
s-ANXA1 in all groups at admission (**A**) and in COVID-19 patient groups throughout hospitalization (**B**). The period under dexamethasone’s biological effect is also depicted in B. Results are presented in a Box-and-Whiskers plot. Controls (*n* = 23); severe: admission (*n* = 27), days 3–4 (*n* = 19), days 5–8 (*n* = 15), week 2 (*n* = 8), week 3 (*n* = 2), week 4 (*n* = 1); critical: admission (*n* = 17), days 3–4 (*n* = 17), days 5–8 (*n* = 14), week 2 (*n* = 9), week 3 (*n* = 8), week 4 (*n* = 5); critical on VV-ECMO: admission (*n* = 17), days 3–4 (*n* = 17), days 5–8 (*n* = 17), week 2 (*n* = 13), week 3 (*n* = 11), week 4 (*n* = 9). s-ANXA1, serum annexin A1; VV-ECMO, veno-venous extracorporeal membrane oxygenation.

At admission, s-ANXA1 was significantly higher in all groups of COVID-19 patients compared to controls (*p* < 0.010 for severe and critical COVID-19 and *p* < 0.001 for critical COVID-19 on VV-ECMO) (Figure 1A). Despite higher median values in patients with critical COVID-19 on VV-ECMO [19.6 (15.3; 26.9)] compared to severe [13.6 (7.6; 25.8)] and critical patients [13.5 (10.2; 35.0)], ANXA1 was not significantly different between COVID-19 patient groups (Figure 1A).

When comparing the patient groups during hospitalization, we found no significant differences in s-ANXA1 values at any time point (Figure 1B). However, among severe COVID-19 patients, s-ANXA1 concentration significantly increased on days 5–8 and week 2 (*p* < 0.050 vs. Admission), while in critical COVID-19 patients, there was an isolated increase in s-ANXA1 at week 2 (*p* < 0.010 vs. Admission) that returned to admission values at week 3. In critical COVID-19 on VV-ECMO patients, s-ANXA1 values remained unchanged during hospitalization (Figure 1B).

Regarding the dexamethasone-treated COVID-19 patients, s-ANXA1 concentrations did not change with different cumulative doses of the drug (Supplementary Figure S3).

### 3.3. FPR2 at Admission and During Hospitalization

**Figure 2:**
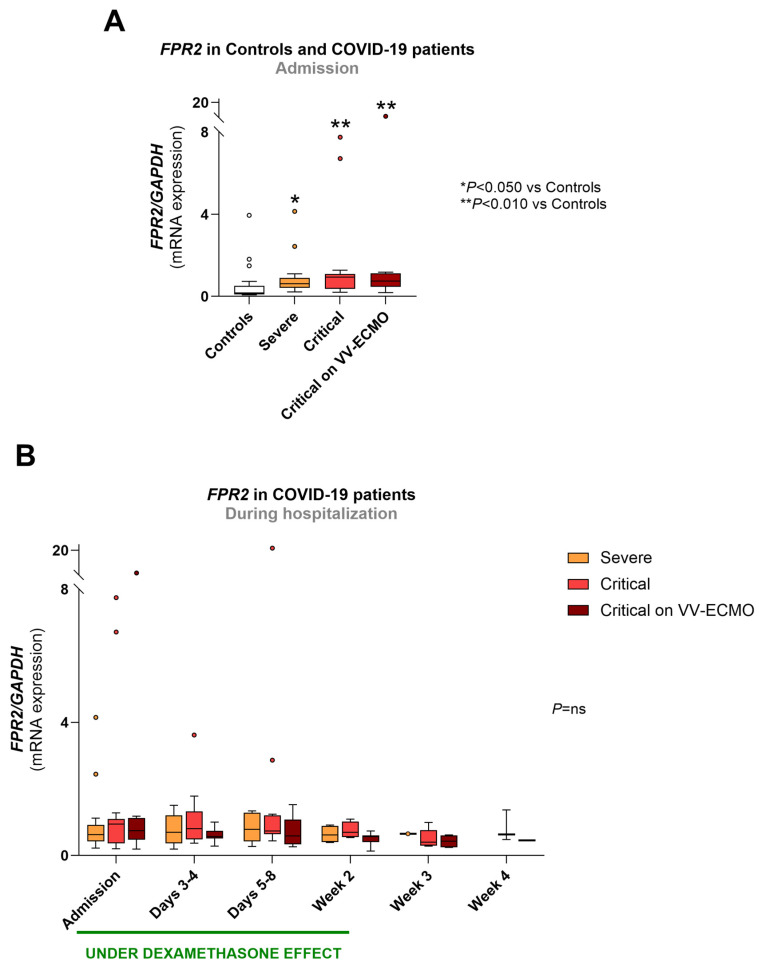
*FPR2* mRNA expression in all groups at admission (**A**) and in COVID-19 patient groups throughout hospitalization (**B**). The period under dexamethasone’s biological effect is also depicted in B. Results are expressed as arbitrary units and presented in Box-and-Whiskers plot. Controls (*n* = 21); severe: admission (*n* = 18), days 3–4 (*n* = 15), days 5–8 (*n* = 11), week 2 (*n* = 4), week 3 (*n* = 1), week 4 (*n* = 0); critical: admission (*n* = 15), days 3–4 (*n* = 13), days 5–8 (*n* = 12), week 2 (*n* = 4), week 3 (*n* = 5), week 4 (*n* = 3); critical on VV-ECMO: admission (*n* = 13), days 3–4 (*n* = 14), days 5–8 (*n* = 14), week 2 (*n* = 10), week 3 (*n* = 4), week 4 (*n* = 2). *FPR2*, FPR2 gene; *GAPDH*, glyceraldehyde 3-phosphate dehydrogenase gene; VV-ECMO, veno-venous extracorporeal membrane oxygenation.

At admission, the mRNA values of *FPR2* were significantly elevated in all groups of COVID-19 patients compared to controls (3.6-fold for severe, 5.4-fold for critical and 4.3-fold for critical on VV-ECMO COVID-19) (Figure 2A).

During hospitalization, *FPR2* mRNA values did not differ between patient groups, nor did they change within each patient group (Figure 2B).

Regarding the COVID-19 patients treated with dexamethasone, *FPR2* mRNA expression did not change with different cumulative doses of the drug (Supplementary Figure S4).

### 3.4. Inflammatory, Endothelial Activation and Additional Proresolving Markers During Hospitalization

To address the secondary objectives, inflammatory, endothelial activation and proresolving parameters were characterized in all patients during hospitalization (Table 2) to enable correlation analyses with ANXA1 and *FPR2*.

Regarding the proinflammatory cytokines evaluated, s-TNF-α concentrations remained unchanged throughout hospitalization in all patient groups. IL-6 significantly decreased only in critical COVID-19 on VV-ECMO patients at week 4 (*p* < 0.050 vs. Admission), while IL-1β showed significant reductions in critical COVID-19 patients at weeks 2 and 3 (*p* < 0.050 vs. Admission). IL-10, a dual role cytokine [57,58], exhibited a decreasing pattern in all patient groups, with significant reductions in severe patients at days 3–4 and 5–8 (*p* < 0.050 and *p* < 0.010 vs. Admission, respectively), in critical patients at days 3–4 (*p* < 0.050) and in critical COVID-19 on VV-ECMO patients at week 3 (*p* < 0.050). s-CRP significantly decreased in severe patients at days 3–4 and 5–8 (*p* < 0.001 vs. Admission) and in critical COVID-19 on VV-ECMO patients at week 4 (*p* < 0.050 vs. Admission).

Among inflammatory cell counts, the total number of leukocytes significantly increased in severe COVID-19 patients at days 5–8 and week 2 (*p* < 0.050 vs. Admission). While neutrophil count did not significantly change throughout hospitalization in any patient group, monocytes and lymphocytes showed significant and consistent increases in both critical groups at multiple time points during hospitalization, in addition to the monocyte elevation observed in severe patients only at week 2 (*p* < 0.050 vs. Admission).

Endothelial activation markers displayed distinct profiles during hospitalization across patient groups. While s-Endocan concentrations significantly decreased only in patients with critical COVID-19 on VV-ECMO at week 4 (*p* < 0.050 vs. Admission), s-VCAM-1 values presented a decreasing pattern in all COVID-19 patients. In contrast, the concentration of s-E-selectin was significantly decreased in severe patients at days 3–4 and 5–8 (*p* < 0.050 vs. Admission) but was increased in critical COVID-19 patients at week 2 (*p* < 0.050 vs. Admission).

With reference to additional proresolving markers, severe COVID-19 patients showed no changes in s-RvD1 or s-RvE1 but presented a significant reduction in *CMKLR1* mRNA expression at days 3–4 and 5–8 admission compared to admission (0.5-fold and 0.4-fold, respectively). On the other hand, a significant decrease was observed for s-RvE1 values in critical COVID-19 patients at days 3–4 (*p* < 0.050 vs. Admission), while s-RvD1 concentrations significantly increased in critical patients at week 3 (*p* < 0.050 vs. Admission) and in critical on VV-ECMO patients at days 5–8 and week 3 (*p* < 0.050 vs. Admission).

### 3.5. Correlation Analysis in All Patients During Hospitalization

**Figure 3:**
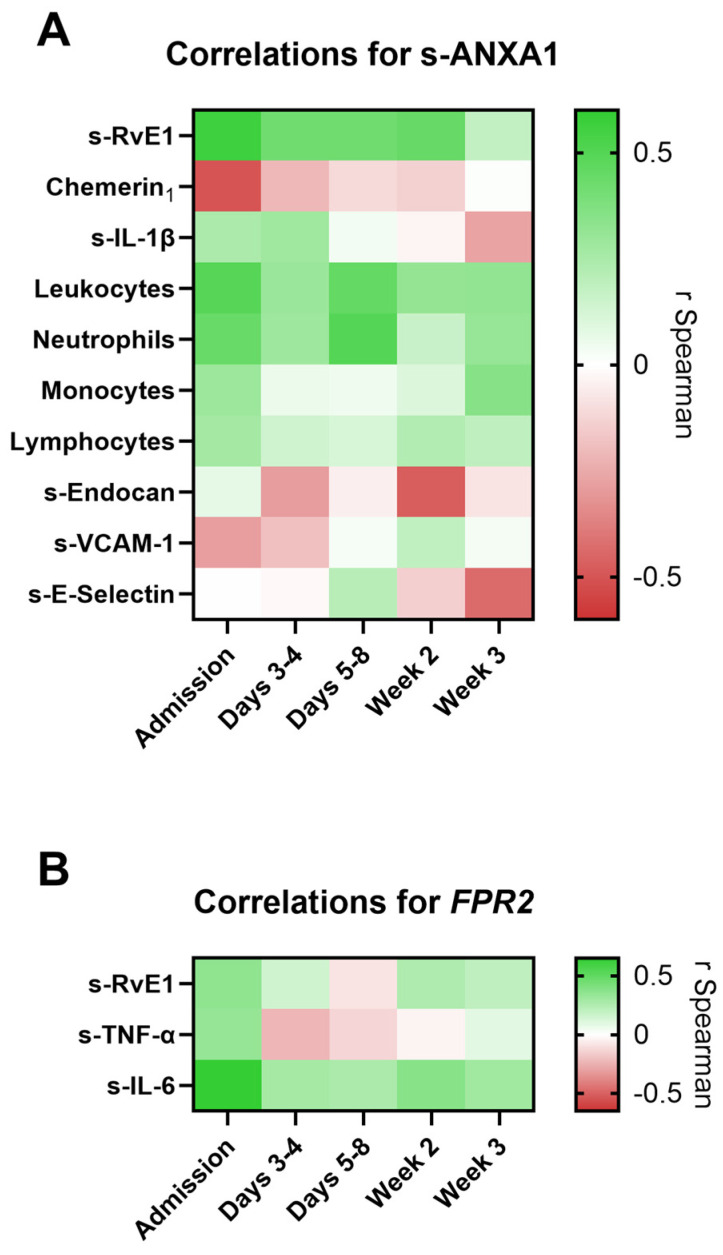
Heatmaps with Spearman correlation coefficients (r Spearman) for s-ANXA1 (**A**) and *FPR2* mRNA expression (**B**) with inflammatory, endothelial and proresolving mediators across hospitalization timepoints. Color gradients indicate the direction and strength of correlations (green, positive; red, negative). Chemerin_1_, chemerin receptor 1; s-ANXA1, serum annexin A1; s-Endocan, serum endocan; s-E-Selectin, serum E-Selectin; s-IL-6, serum interleukin 6; s-IL-1β, serum interleukin 1 beta; s-RvE1, serum resolvin E1. s-TNF-α, serum tumor necrosis factor alpha; s-VCAM-1, serum vascular cell adhesion molecule 1.

Given the exploratory nature of these analyses, correlation patterns between ANXA1/*FPR2* and inflammatory, endothelial and proresolving parameters across hospitalization timepoints are presented as heatmaps with correlation coefficients (Figure 3). Detailed Spearman correlation coefficients and adjusted *p*-values are provided in the Supplementary Table S2.

There was a consistent positive correlation between s-ANXA1 and s-RvE1 concentrations from admission until week 2. Additionally, an inverse correlation pattern between s-ANXA1 and the RvE1 receptor, Chemerin_1_, was observed. Moreover, s-ANXA1 exhibited positive correlations with inflammatory factors, namely the proinflammatory cytokine s-IL-1β, and with inflammatory cell counts, such as total leukocytes, neutrophils, lymphocytes and monocytes. Conversely, mainly inverse correlations were observed between s-ANXA1 and endothelial dysfunction markers throughout hospitalization, including with s-Endocan, s-VCAM-1 and s-E-selectin (Figure 3A).

*FPR2* was positively correlated at admission with s-RvE1, as well as with proinflammatory cytokines, such as s-TNF-α and s-IL-6 (Figure 3B). The positive correlation with s-IL-6 persisted throughout hospitalization, although it reached statistical significance only at admission (Supplementary Table S2). 

### 3.6. ANXA1 and FPR2 in Survivors Versus Non-Survivors COVID-19 Patients

**Figure 4:**
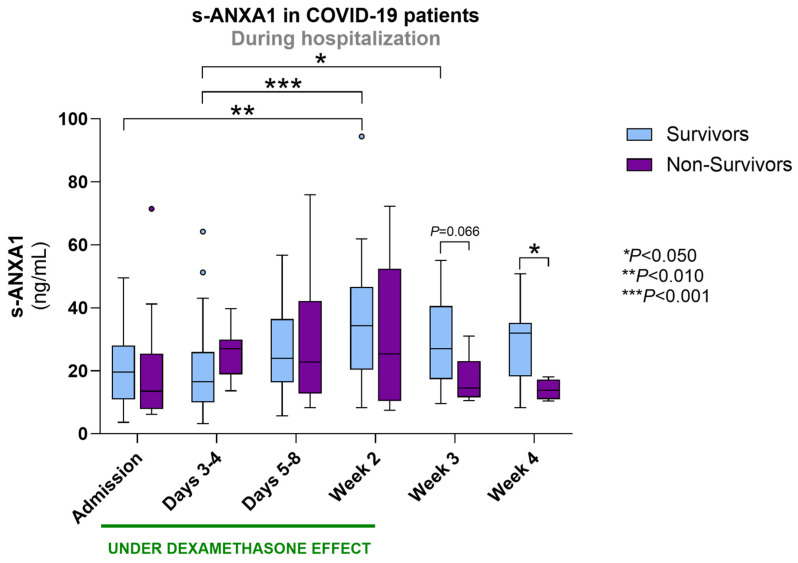
s-ANXA1 in COVID-19 survivors and non-survivors throughout hospitalization (intrahospital mortality). The period under dexamethasone’s biological effect is also depicted. Results are presented in a Box-and-Whiskers plot. Survivors: admission (*n* = 49), days 3–4 (*n* = 43), days 5–8 (*n* = 36), week 2 (*n* = 24), week 3 (*n* = 15), week 4 (*n* = 10); non-survivors: admission (*n* = 11), days 3–4 (*n* = 9), days 5–8 (*n* = 9), week 2 (*n* = 5), week 3 (*n* = 5), week 4 (*n* = 4). s-ANXA1, serum annexin A1.

When comparing s-ANXA1 between surviving and non-surviving COVID-19 patients during hospitalization, we observed higher concentrations in survivors at weeks 3 and 4, with a statistically significant difference at week 4 (*p* < 0.050). In fact, COVID-19 patients who survived presented a progressive increase in s-ANXA1 values throughout hospitalization, which became significant at week 2 (*p* < 0.010 vs. Admission; *p* < 0.001 vs. Days 3–4) and week 3 (*p* < 0.050 vs. Days 3–4). In contrast, non-survivors did not exhibit significant changes in s-ANXA1 values over time (Figure 4).

**Figure 5:**
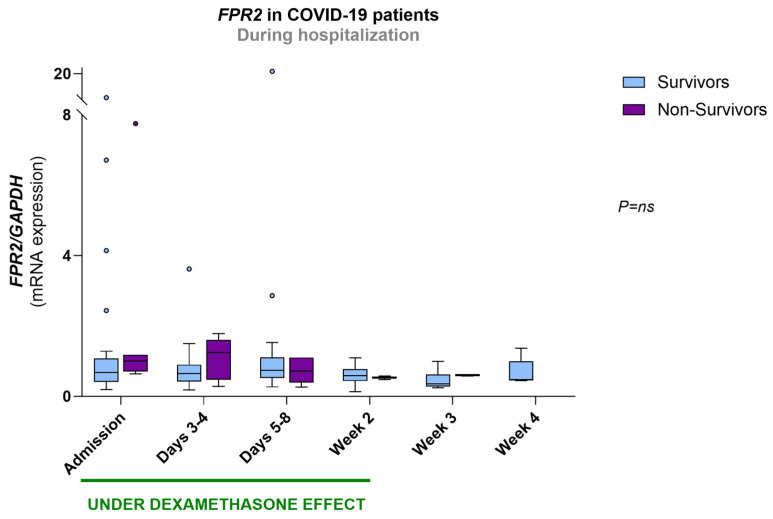
*FPR2* mRNA expression in COVID-19 survivors and non-survivors throughout hospitalization (intrahospital mortality). The period under dexamethasone’s biological effect is also depicted. Results are presented in a Box-and-Whiskers plot. Survivors: admission (*n* = 38), days 3–4 (*n* = 35), days 5–8 (*n* = 31), week 2 (*n* = 15), week 3 (*n* = 8), week 4 (*n* = 5); non-survivors: admission (*n* = 7), days 3–4 (*n* = 6), days 5–8 (*n* = 5), week 2 (*n* = 2), week 3 (*n* = 2), week 4 (*n* = 0). *FPR2*, FPR2 gene; *GAPDH*, glyceraldehyde 3-phosphate dehydrogenase gene.

*FPR2* mRNA expression showed no significant differences between survivors and non-survivors at any time point and remained stable within each group throughout hospitalization (Figure 5).

### 3.7. Repeated Measures Multivariate Analysis for s-ANXA1 in All COVID-19 Patients

During the hospitalization period from admission until week 2, no significant associations were found between s-ANXA1 and the critical groups (Model 1: Critical—Adjusted β: 2.42; *p* = 0.486; Critical on VV-ECMO—Adjusted β: 3.31; *p* = 0.296) nor with intrahospital mortality (Model 3: Adjusted β: −1.87; *p* = 0.624) (Table 3). However, s-ANXA1 showed a significant positive association with s-RvE1 (Model 2: Adjusted β: 0.019; *p* < 0.001) (Table 3).

When considering only the data from weeks 3 and 4, we still did not find a significant association of s-ANXA1 with intrahospital mortality (Model 4: Adjusted β: 8.48; *p* = 0.131) (Table 3). However, the adjusted β-value shifted from negative in the earlier period (from admission to week 2) (Model 3: β: −1.87) to positive in the later phase (weeks 3 and 4) (Model 4: β: 8.48) (Table 3).

Interestingly, female sex was significantly associated with lower values of s-ANXA1 throughout hospitalization (admission to week 4) when adjusted for age and intrahospital mortality (Model 3: Adjusted β: −5.29; *p* = 0.036; Model 4: Adjusted β: −9.97; *p* = 0.010) (Table 3).

## 4. Discussion

Our findings reveal that all COVID-19 patient groups exhibited elevated circulating ANXA1 and increased *FPR2* expression upon hospital admission. Moreover, although ANXA1 concentrations were initially similar across severity groups and between survivors and non-survivors, only severe COVID-19 patients and survivors presented a sustained increase over time, persisting even after the end of dexamethasone treatment. Furthermore, although both ANXA1 and *FPR2* were positively correlated with inflammation, ANXA1 presented inverse correlations with several endothelial activation markers throughout hospitalization. Interestingly, ANXA1 and RvE1 exhibited a consistent positive association in all COVID-19 patients throughout the first two weeks of hospitalization, which coincided with the period under dexamethasone effects.

Glucocorticoids are immunomodulatory agents that modulate immune cell activity and the balance of pro- and anti-inflammatory mediators through the regulation of gene expression and intracellular signaling [59]. In COVID-19, multi-omics analyses have shown their ability to modulate dysregulated pathways, namely neutrophil and platelet activation [60]. Several clinical trials, most notably the RECOVERY trial, demonstrated that glucocorticoids reduce mortality and hospitalization time in severe and critical COVID-19 patients without significant adverse effects [61,62,63]. In contrast, no benefit or harm was observed in patients without respiratory support [61,64]; thus, glucocorticoids are not recommended in non-severe cases [13,14]. Given their clinical efficacy in COVID-19 and the established role of the ANXA1-FPR2 pathway in mediating some of the glucocorticoids’ effects, as evidenced by the attenuated drug response in ANXA1-deficient animals [65,66], this pathway likely plays a significant role in COVID-19 disease and warrants further investigation. In our cohort, nearly all COVID-19 patients began dexamethasone treatment upon hospital admission, which may explain the upregulation of both ANXA1 values and *FPR2* mRNA expression compared to controls. However, as all blood samples were collected after treatment initiation, the specific effect of dexamethasone could not be assessed. However, the exposure to different cumulative doses of dexamethasone did not appear to significantly impact ANXA1 concentrations and *FPR2* mRNA values. On the other hand, the rise in ANXA1 could also be driven by the inflammatory environment, as studies have shown that neutrophil activation promotes its release from cells [22], and IL-6 and lipopolysaccharide stimulate its expression, which supports the relevance of ANXA1 as an acute phase protein [67,68]. Likewise, *FPR2* expression is upregulated by various cytokines, such as TNF-α, IL-13 and IFN-γ [69,70]. Our results further support this, since ANXA1 positively correlated with inflammatory cells and IL-1β, and *FPR2* showed positive correlations with IL-6 and TNF-α. However, this study cannot separate the effects of dexamethasone from endogenous inflammatory signaling on ANXA1-FPR2, and the observed patterns likely reflect both influences. Accordingly, two other studies reported increased ANXA1 concentrations in all COVID-19 patients at hospital presentation compared to healthy controls [51,52]. Also, Busch et al. observed significant positive correlations of ANXA1 with inflammatory markers [52], although these studies did not assess FPR2, and dexamethasone use was either absent or not documented [51,52]. Conversely, Koenis et al. evaluated *FPR2* expression in different immune cells and found no differences between COVID-19 patients and controls [9]. These differences may also result from the fact that FPR2 interacts with other structurally diverse and functionally contrasting molecules, and its expression can be influenced by the contributions of all ligands [25,71]. Although ANXA1 signaling through FPR1 has been proposed to contribute to excessive inflammation in severe COVID-19 patients [72], only the synthetic Ac2-26 peptide derived from the N-terminal domain of ANXA1 has been demonstrated to interact with FPR1 in addition to FPR2 [73,74]. Given that full-length ANXA1 selectively activates FPR2 [74], this study focused on *FPR2* expression, and FPR1 was not assessed.

In our cohort, ANXA1 levels did not significantly differ between severity groups, although critical COVID-19 patients on VV-ECMO presented higher median concentrations at the beginning of the hospitalization period. Similarly, Busch et al. found no differences in ANXA1 across hospitalized patients with varying disease severity, speculating that the most severe patients may have exceeded their capacity to secrete ANXA1 [52]. Nevertheless, they reported significantly higher ANXA1 concentrations in the most severe cases compared to non-hospitalized individuals [52], a patient group not included in our study. Here, dexamethasone treatment may have masked potential ANXA1 differences inherently linked to disease severity. In contrast, Canacik et al. and Shenoy et al. demonstrated a negative association between ANXA1 values and COVID-19 disease severity [49,50]. Again, none of these studies mentioned dexamethasone treatment [49,50]. Furthermore, we observed no differences in *FPR2* mRNA expression across disease severity, consistent with the findings of Koenis et al. [9].

Importantly, we also analyzed the longitudinal profiles of ANXA1 and its receptor throughout hospitalization, comparing them across severity groups and survival outcomes. While *FPR2* mRNA expression remained stable from admission, ANXA1 significantly increased in severe COVID-19 patients and survivors, maintaining elevated levels even after dexamethasone treatment ended. In contrast, this pattern was not observed in critical patients and non-survivors. Notably, non-survivors presented significantly lower ANXA1 levels compared to survivors at later stages of hospitalization. This raises the possibility that less severe patients and survivors, unlike the critically ill and non-survivors, may have been able to maintain ANXA1 production even beyond the dexamethasone treatment period, potentially counteracting hyperinflammation, promoting resolution and contributing to clinical improvement. Busch et al. reported a similar finding, noting that patients without adverse outcomes exhibited a greater increase in ANXA1 concentration over time [52]. Furthermore, although the associations of ANXA1 with mortality did not reach statistical significance in the multivariate analysis adjusted for age and sex, the adjusted β-value shifted from negative during the initial period of hospitalization (admission to week 2) to positive in weeks 3 and 4, suggesting a possible positive association between ANXA1 levels and survival only during the recovery phase. However, the findings at later timepoints are limited by the small sample size and may reflect random variation, requiring cautious interpretation and validation in larger cohorts to confirm the prognostic value of ANXA1 in COVID-19. Studies investigating the molecular signatures of recovered COVID-19 patients have shown an upregulation of ANXA1 in monocytes, corroborating a potential role for ANXA1 in the recovery phase of the disease [6,75]. This underscores the promising therapeutic potential of ANXA1 activation in COVID-19, as previously proposed, given the demonstrated benefits of ANXA1 or its mimetic peptide administration in multiple experimental models of lung injury and other organs impacted by the disease [76,77,78,79]. Notably, Ac2-26 administration in SARS-CoV-2-infected mice protected against lethal outcomes without impairing viral clearance [37].

ANXA1 is known to control leukocyte recruitment to inflamed tissues by preventing their adhesion to, and migration across, blood vessels [80]. Mechanistically, it appears to modulate leukocyte–endothelium interactions by interfering with adhesion molecules. For instance, treatment with ANXA1 or its mimetic peptide promoted L-selectin shedding in neutrophils [81,82]. In another study, ANXA1 interacted with monocytes α4β1-integrin, preventing its binding to VCAM-1 in endothelial cells [83]. Moreover, studies on human endothelial cells have shown that both exogenous Ac2-26 and endogenous ANXA1 suppress TNF-α-induced upregulation of intercellular adhesion molecule 1 (ICAM-1), VCAM-1 and E-selectin [19,84]. Consistent with these findings, our study revealed inverse correlations between ANXA1 and endothelial adhesion molecules (VCAM-1 and E-selectin), as well as endocan, a proteoglycan secreted by activated endothelium [85], in all patients throughout hospitalization. This further supports ANXA1’s ability to modulate endothelial function and its potential role in mitigating COVID-19 endotheliitis, a phenomenon previously explored by our group [53].

In addition to ANXA1, other proresolving mediators can reduce endothelial activation and leukocyte infiltration [86,87]. While these and other overlapping actions suggest a potential crosstalk between proresolving molecules, this putative network remains largely unexplored. Brancaleone et al. reported an interaction between LXA_4_ and ANXA1, showing that LXA_4_ induces ANXA1 mobilization in neutrophils [26]. Additionally, they demonstrated that the antimigratory effects of LXA_4_ were lost in ANXA1-deficient mice, proposing ANXA1 as a central player in an endogenous network via FPR2 [26]. An increase in ANXA1 expression was also reported in obese mice treated with LXA_4_ [28]. Similarly, RvE1, an SPM acting independently of FPR2, has also been shown to induce LXA_4_ production in vivo [31]. Strengthening the evidence of an interconnected network among proresolving mediators, we demonstrated here a consistent positive correlation between ANXA1 and RvE1 from admission up to the second week of hospitalization. This association, further confirmed by multivariate analysis, suggests a coordinated role of these mediators in counteracting hyperinflammation. Moreover, at admission, *FPR2* positively correlated with RvE1, while ANXA1 showed an inverse correlation with Chemerin_1_. In fact, in this cohort of COVID-19 patients, the RvE1 receptor displayed an opposite pattern to its ligand [54]. One common feature of ANXA1 and some SPMs is their dependency on calcium levels. ANXA1 requires calcium for membrane binding and N-terminal exposure to engage its receptor [88], and the production of lipoxin and D- and E-series resolvins also relies on calcium, which enables the activation of their biosynthetic enzyme, 5-lipoxygenase, by its activating protein, FLAP [89]. Moreover, ANXA1-induced FPR2 activation triggers an intracellular calcium increase [82]. Hence, this could serve as a potential link between these endogenous mediators of resolution, facilitating their interplay. However, more research is needed to uncover the distinct mechanisms governing each mediator’s interactions.

Interestingly, multivariate analysis adjusted for age and intrahospital mortality revealed that female patients were associated with lower concentrations of ANXA1 compared to males. To date, the influence of sex on ANXA1 levels in COVID-19 patients remains unexplored. However, both in vitro and in vivo studies suggest that estrogens, such as 17β-estradiol and its metabolite 2-methoxyestradiol, upregulate ANXA1 expression, implying potentially higher levels in females [90,91,92]. Supporting this, Nadkarni et al. reported that polymorphonuclear cells from pre-menopausal women expressed more ANXA1 than those from men [93]. Nonetheless, the dramatic decline in estrogen levels following menopause is associated with a reduction in ANXA1 expression, as demonstrated in vascular tissues of female mice from 2 to 15 months of age and in ovariectomized animals [90,94]. This may explain the lower levels of ANXA1 observed in the female patients of our cohort, whose median age was 63 (57; 70) years, but larger studies are required to validate these findings.

This is the first study to evaluate both ANXA1 and its receptor throughout hospitalization of COVID-19 patients and the first to include a group of patients on VV-ECMO. Additionally, we paved the way for investigating associations between proresolving mediators in humans, revealing a potential interaction that warrants future exploration both in vitro and in vivo.

Nevertheless, our study has certain limitations. First, it includes a small sample size and a single-center design, which could lead to selection bias due to consecutive recruitment. Second, blood samples were not always collected at all time points for every patient due to the overwhelming clinical demands during the COVID-19 pandemic and patient withdrawal of consent often stemming from fear and psychological distress experienced by hospitalized COVID-19 patients. Additionally, the marked decrease in sample availability at later time points (Supplementary Figure S1) may introduce survivor bias because some non-survivor patients were not present in the analyses at these time points. Nevertheless, the patients who remained in the study at later time points were predominantly those from the critical groups, as they required prolonged hospitalization. As a result, these findings mainly reflect the characteristics of critically ill patients. Third, limited access to clinical data for the control group precluded assessment of comorbidities, medications, and baseline inflammatory status. However, all donors underwent clinical evaluation at the time of blood donation to exclude acute disease. Fourth, the ELISA kit used for ANXA1 quantification does not distinguish between the intact and cleaved forms, possibly measuring both. Notably, studies have reported ANXA1 cleavage following leukocyte activation [95,96,97]. In addition, the cleaved form has been suggested to exhibit proinflammatory properties [98], with elevated levels detected in human and animal inflammatory conditions [99,100,101], including in SARS-CoV-2-infected mice [37]. However, studies by Vago et al. and Tavares et al. suggest that the cleaved form of ANXA1 is predominant only during the acute phase of inflammation, while the intact form prevails in the resolution phase [100,101]. Applying these findings to our study, the rise in ANXA1 concentration at admission across all COVID-19 patients could primarily reflect the cleaved form, whereas the later increase observed only in severe patients and survivors may mainly correspond to the intact form. Interestingly, dexamethasone seems to prevent ANXA1 cleavage [100], further supporting this assumption. However, this distinction can only be confirmed by measuring both ANXA1 forms separately. Fifth, our analysis was limited to PBMC-derived mRNA expression due to insufficient PMN recovery, hampering the evaluation of all FPR2-expressing immune cells, and future studies employing protein-based methods to assess receptor surface expression would provide valuable complementary data. Finally, the use of ELISA kits for resolvin quantification is also a limitation, as this method may present some cross-reactivity with structurally similar molecules, such as SPM precursors or metabolites. Currently, the gold standard for SPMs analysis is LC/MS-MS, as previously discussed [54,89]. Nevertheless, since LC/MS-MS requires costly equipment and highly specialized personnel, the quantification of SPMs by ELISA kits remains widely used in clinical studies [79,102,103,104]. Furthermore, there is an ongoing discussion regarding SPM quantification by LC/MS-MS, because there is considerable interlaboratory variability in SPM concentrations evaluated in the serum and plasma samples from healthy controls, likely influenced by factors such as sample storage, processing methods, differences in equipment sensitivity and contrasting approaches to data analysis and integration [105,106,107].

## 5. Conclusions

Our findings suggest the involvement of ANXA1 in the COVID-19 recovery phase, reinforcing its contribution to the positive clinical outcomes of glucocorticoid therapy in hospitalized patients. Nevertheless, the development of novel agents lacking the adverse effects associated with this class of drugs is urgently needed. ANXA1 N-terminal mimetic peptides or cleavage-resistant variants emerge as promising alternatives, warranting further investigation in experimental models and human studies. Additionally, we identified a potential interplay between ANXA1 and RvE1 in COVID-19 patients, highlighting the therapeutic advantage of promoting inflammation resolution to amplify multiple beneficial pathways in this process.

## Figures and Tables

**Table 1:** Demographic and clinical characterization at admission and follow-up parameters of the study population.

Demographic and Clinical Parameters	Controls (*n* = 23)	Severe COVID-19 (*n* = 27)	Critical COVID-19 (*n* = 17)	Critical COVID-19 on VV-ECMO (*n* = 17)	*p* Value
**Age (Years)**	**57 (53; 63)**	71 (63; 80) **	**67 (55; 72)**	**55 (40; 59) ^###,$^**	**<0.001**
Sex: Men, *n* (%)	15 (65)	17 (63)	11 (65)	11 (65)	0.999
Sex: Women, *n* (%)	8 (35)	10 (37)	6 (35)	6 (35)	0.999
Comorbidities, *n* (%)					
Diabetes	n.d.	11 (41)	6 (35)	4 (24)	0.502
Obesity	n.d.	7 (26)	8 (47)	10 (59)	0.081
Arterial Hypertension	n.d.	18 (67)	13 (76)	8 (47)	0.188
Heart Failure	n.d.	6 (22)	3 (18)	1 (6)	0.357
Respiratory Disease	n.d.	8 (30)	4 (24)	2 (12)	0.389
Renal Disease	n.d.	6 (22)	4 (24)	0 (0)	0.099
Malignancy	n.d.	2 (7)	0 (0)	0 (0)	0.272
APACHE II Score	n/a	n/a	17 ± 2	19 ± 2	0.423
SAPS II Score	n/a	n/a	42 ± 4	40 ± 4	0.666
Therapeutics at Admission, *n* (%)					
Dexamethasone	n/a	21 (78)	16 (94)	16 (94)	0.172
Remdesivir	n/a	1 (4)	0 (0)	2 (12)	0.263
Antibiotics	n/a	5 (19)	7 (41)	9 (53)	0.051
**PaO_2_/FiO_2_ ratio**	n/a	**257 (230; 287)**	**92 (68; 137) ^###^**	**100 (76; 119) ^###^**	**<0.001**
**PaCO_2_ (mmHg)**	n/a	**32 ± 1**	**37 ± 1 ^#^**	**48 ± 2 ^###,$$$^**	**<0.001**
Lactate (mmol/L)	n/a	1.1 (1.0; 1.6)	1.5 (1.1; 1.8)	1.5 (1.2; 1.7)	0.258
Follow-up					
Type of Oxygen Support During Hospitalization, *n* (%)					
** Mechanical Ventilation**	n/a	**2 (7)**	**11 (65)**	**17 (100)**	**<0.001**
** NIV**	n/a	**5 (19)**	**11 (65)**	**14 (82)**	**<0.001**
** High-Flow Cannula**	n/a	**9 (33)**	**13 (76)**	**9 (53)**	**0.020**
Supplementary Oxygen	n/a	26 (96)	13 (76)	16 (94)	0.081
** Length of ICU stay (days)**	n/a	**0 (0; 0)**	**16 (7; 33) ^###^**	**34 (16; 74) ^###^**	**<0.001**
** Total length of hospital stay (days)**	n/a	**7 (5; 15)**	**22 (11; 57) ^##^**	**43 (25; 116) ^###^**	**<0.001**
Intrahospital mortality, *n* (%)	n/a	3 (11)	4 (24)	4 (25)	0.423

Some data were previously published in other studies within the main research project [12,53,54]. APACHE II, acute physiology and chronic health evaluation II; FiO_2_, fraction of inspired oxygen; ICU, Intensive Care Unit; n/a, not applicable; n.d., not determined; NIV, non-invasive ventilation; PaCO_2_, partial pressure of arterial carbon dioxide; PaO_2_, Partial pressure of arterial oxygen; SAPS II, Simplified Acute Physiology Score II; VV-ECMO, veno-venous extracorporeal membrane oxygenation; Results are expressed as number (%), mean ± SEM or as median (25th percentile; 75th percentile) for data with normal or non-normal distribution, respectively. ** *p* < 0.010 vs. Controls; ^#^ *p* < 0.050 vs. Severe; ^##^ *p* < 0.010 vs. Severe; ^###^ *p* < 0.001 vs. Severe; ^$^ *p* < 0.050 vs. Critical; ^$$$^ *p* < 0.001 vs. Critical. Parameters with significant differences between groups are highlighted in bold.

**Table 2:** Inflammatory, endothelial activation and additional proresolving markers in patients with severe COVID-19, critical COVID-19 and critical COVID-19 on VV-ECMO throughout hospitalization.

**Severe COVID-19**						
	**Admission**	**Days 3–4**	**Days 5–8**	**Week 2**	**Week 3**	**Week 4**
Proinflammatory cytokines						
s-TNF-α (pg/mL)	19.9 (13.2; 31.5)	18.2 (13.5; 27.9)	20.9 (15.6; 24.0)	20.6 (14.1; 27.5)	34.9 (16.1; 53.8)	26.4 (26.4; 26.4)
s-IL-6 (pg/mL)	8.4 (5.2; 19.0)	7.8 (5.0; 20.8)	10.3 (4.2; 17.7)	18.4 (8.5; 37.1)	50.1 (44.4; 55.8)	3.4 (3.4; 3.4)
s-IL-1β (pg/mL)	0.9 (0.3; 1.5)	0.9 (0.5; 1.5)	1.2 (0.5; 2.6)	0.5 (0.05; 0.9)	0.5 (0.5; 0.5)	0.2 (0.2; 0.2)
Dual role cytokine						
s-IL-10 (pg/mL)	37.5 (18.8; 76.5)	26.4 (23.8; 48.4) *	28.3 (17.1; 40.9) **	15.0 (6.7; 50.8)	23.1 (15.3; 30.8)	5.7 (5.7; 5.7)
Inflammatory marker						
s-CRP (mg/L)	100 (48; 173)	47 (14; 63) ***	20 (4; 58) ***	16 (6; 158)	149 (100; 198)	69 (69; 69)
Inflammatory cells						
Leukocytes (×10^9^/L)	6 (5; 11)	7 (5; 10)	9 (7; 11) *	9 (9; 13) *	12 (10; 14)	7 (7; 7)
Neutrophils (×10^9^/L)	5 (3; 9)	6 (4; 9)	7 (5; 9)	7 (7; 9)	9 (6; 11)	5 (5; 5)
Monocytes (×10^9^/L)	0.4 (0.3; 0.6)	0.5 (0.4; 0.6)	0.5 (0.3; 0.7)	0.9 (0.6; 1.0) *	1.3 (0.8; 1.8)	0.5 (0.5; 0.5)
Lymphocytes (×10^9^/L)	0.9 (0.6; 1.3)	0.9 (0.7; 1.3)	0.9 (0.8; 2.0)	1.3 (0.7; 3.7)	1.2 (1.0; 1.4)	0.9 (0.9; 0.9)
Endothelial activation markers						
s-Endocan (ng/mL)	3.4 (2.3; 5.1)	5.2 (3.0; 7.5)	4.4 (2.5; 11.2)	4.6 (3.9; 6.5)	5.4 (5.3; 5.6)	3.2 (3.2; 3.2)
s-VCAM-1 (ng/mL)	5828 (2705; 7054)	4463 (2080; 7362) *	4385 (2356; 7385)	3852 (2684; 6072)	3883 (1463; 6303)	3582 (3582; 3582)
s-E-Selectin (ng/mL)	32.3 (26.4; 40.9)	29.0 (20.5; 46.5) *	25.7 (18.7; 42.9) *	33.2 (27.4; 39.2)	39.1 (27.8; 50.4)	27.5 (27.5; 27.5)
SPMs						
s-RvD1 (pg/mL)	90.9 (80.3; 111.2)	85.9 (76.5; 95.3)	82.4 (76.6; 100.2)	95.6 (88.2; 106.4)	96.6 (83.2; 110.0)	79.0 (79.0; 79.0)
s-RvE1 (pg/mL)	989 ± 81	1071 ± 99	987 ± 82	1045 ± 127	1106 ± 202	972 ± 0
RvE1 receptor						
*CMKLR1/GAPDH* (mRNA expression)	2.5 (1.1; 3.4)	1.2 (1.0; 1.7) *	1.1 (1.0; 1.5) *	1.7 (1.5; 2.5)	1.0 (1.0; 1.0)	-
**Critical COVID-19**						
	**Admission**	**Days 3–4**	**Days 5–8**	**Week 2**	**Week 3**	**Week 4**
Proinflammatory cytokines						
s-TNF-α (pg/mL)	26.3 (18.8; 37.3)	23.0 (15.2; 42.5)	30.8 (21.4; 47.9)	45.4 (38.4; 55.5)	35.2 (20.4; 59.5)	39.4 (33.5; 64.7)
s-IL-6 (pg/mL)	15.4 (5.7; 51.9)	14.5 (4.2; 27.4)	18.5 (4.5; 55.0)	40.1 (24.9; 165.4)	26.8 (7.4; 71.7)	102.3 (17.5; 192.4)
s-IL-1β (pg/mL)	1.7 (1.2; 2.5)	1.8 (0.9; 2.8)	1.5 (1.2; 1.9)	1.1 (0.3; 1.9) *	1.4 (0.5; 1.7) *	0.9 (0.9; 2.3)
Dual role cytokine						
s-IL-10 (pg/mL)	50.6 (31.9; 85.2)	33.7 (18.9; 53.1) *	43.6 (24.0; 96.7)	34.5 (14.2; 62.2)	16.1 (7.5; 29.1)	11.9 (4.5; 34.8)
Inflammatory marker						
s-CRP (mg/L)	116 (78; 190)	69 (21; 134)	91 (19; 128)	157 (58; 244)	99 (38; 211)	87 (54; 241)
Inflammatory cells						
Leukocytes (×10^9^/L)	9 (6; 12)	8 (7; 11)	10 (8; 12)	15 (10; 17)	8 (5; 10)	7 (6; 9)
Neutrophils (×10^9^/L)	9 (6; 12)	7 (5; 9)	9 (6; 11)	11 (6; 14)	6 (3; 8)	5 (4; 6)
Monocytes (×10^9^/L)	0.4 (0.3; 0.6)	0.5 (0.3; 0.7) *	0.5 (0.4; 0.7)	0.7 (0.6; 1.1) *	0.6 (0.4; 0.7) *	0.8 (0.4; 1.0)
Lymphocytes (×10^9^/L)	0.7 (0.5; 1.1)	0.7 (0.5; 1.1)	0.7 (0.6; 1.2)	1.3 (0.7; 1.7) *	1.2 (1.0; 1.5) *	1.0 (0.8; 1.7)
Endothelial activation markers						
s-Endocan (ng/mL)	3.3 (1.8; 5.6)	3.7 (2.9; 6.1)	5.9 (3.3; 9.7)	7.5 (3.3; 10.9)	4.2 (2.5; 5.8)	4.0 (2.6; 5.7)
s-VCAM-1 (ng/mL)	4416 (2502; 5986)	3617 (2401; 4574) *	3168 (2525; 4179) *	2786 (2433; 5221)	2398 (1510; 4127)	1743 (1533; 4898)
s-E-Selectin (ng/mL)	34.5 (22.2; 45.3)	35.5 (23.3; 45.9)	39.8 (22.3; 63.6)	59.3 (30.5; 81.9) *	55.0 (42.2; 68.3)	48.7 (41.7; 82.9)
SPMs						
s-RvD1 (pg/mL)	79.9 (68.4; 97.2)	77.4 (64.2; 88.2)	85.2 (72.0; 97.7)	90.6 (69.3; 98.0)	101.4 (83.1; 107.8) *	97.5 (91.7; 126.4)
s-RvE1 (pg/mL)	1247 ± 116	1056 ± 87 *	1034 ± 126	1403 ± 130	1049 ± 75	972 ± 139
RvE1 receptor						
*CMKLR1/GAPDH* (mRNA expression)	0.7 (0.3; 1.5)	0.6 (0.4; 0.9)	0.5 (0.3; 1.2)	0.5 (0.3; 1.0)	1.3 (0.6; 1.8)	1.2 (0.9; 1.3)
**Critical COVID-19 on VV-ECMO**						
	**Admission**	**Days 3–4**	**Days 5–8**	**Week 2**	**Week 3**	**Week 4**
Proinflammatory cytokines						
s-TNF-α (pg/mL)	21.8 (14.3; 30.0)	27.8 (19.4; 37.9)	37.3 (22.0; 49.2)	32.9 (24.3; 40.6)	31.5 (14.6; 47.6)	27.8 (16.8; 34.9)
s-IL-6 (pg/mL)	27.4 (4.1; 142.7)	25.5 (17.3; 40.4)	31.1 (14.8; 78.4)	25.0 (16.4; 77.0)	24.9 (9.5; 34.6)	8.2 (5.8; 39.3) *
s-IL-1β (pg/mL)	1.3 (0.8; 2.7)	1.8 (0.6; 4.1)	1.8 (0.3; 3.2)	1.9 (1.0; 7.5)	1.5 (0.0; 2.7)	1.4 (0.6; 3.6)
Dual role cytokine						
s-IL-10 (pg/mL)	31.8 (21.5; 67.3)	50.4 (25.1; 66.8)	25.4 (14.8; 45.3)	22.4 (18.6; 42.0)	22.1 (12.2; 31.6) *	26.6 (12.7; 44.8)
Inflammatory marker						
s-CRP (mg/L)	163 (116; 245)	101 (52; 237)	115 (43; 182)	91 (53; 169)	116 (64; 189)	97 (55; 205) *
Inflammatory cells						
Leukocytes (×10^9^/L)	12 (9; 13)	13 (11; 18)	13 (10; 17)	13 (9; 17)	10 (8; 11)	9 (7; 14)
Neutrophils (×10^9^/L)	10 (8; 12)	10 (9; 15)	10 (7; 12)	10 (6; 12)	7 (4; 8)	6 (4; 9)
Monocytes (×10^9^/L)	0.6 (0.3; 0.8)	0.5 (0.3; 0.9)	0.8 (0.4; 1.1)	0.9 (0.6; 1.1) **	0.9 (0.6; 1.2) **	0.8 (0.6; 1.1) *
Lymphocytes (×10^9^/L)	1.0 (0.7; 1.5)	1.2 (0.7; 1.8)	1.5 (1.1; 2.1) *	2.0 (1.4; 2.9) **	1.6 (1.2; 2.1) **	1.9 (1.0; 2.6)
Endothelial activation markers						
s-Endocan (ng/mL)	6.1 (5.4; 9.8)	5.0 (4.3; 8.7)	6.2 (4.7; 7.7)	5.3 (3.1; 6.7)	5.7 (3.6; 6.3)	4.6 (3.7; 6.1) *
s-VCAM-1 (ng/mL)	3025 (1809; 3762)	2997 (1744; 3795)	2780 (1621; 3306)	1479 (1220; 2724) *	1598 (1385; 2559) **	1943 (1274; 2800) *
s-E-Selectin (ng/mL)	40.6 (34.3; 58.5)	39.7 (33.8; 51.5)	38.1 (33.9; 54.1)	45.1 (38.5; 53.7)	43.8 (35.3; 50.5)	45.1 (29.3; 48.3)
SPMs						
s-RvD1 (pg/mL)	82.2 (66.8; 101.3)	73.6 (67.4; 101.9)	86.9 (74.6; 103.9) *	89.6 (74.4; 124.0)	112.2 (78.8; 144.0) *	112.3 (75.4; 126.4)
s-RvE1 (pg/mL)	1311 ± 76	1368 ± 65	1379 ± 73	1257 ± 81	1153 ± 88	1328 ± 125
RvE1 receptor						
*CMKLR1/GAPDH* (mRNA expression)	0.5 (0.1; 0.8)	0.5 (0.2; 0.8)	0.5 (0.3; 0.7)	0.6 (0.3; 1.0)	2.0 (1.1; 2.7)	1.6 (1.3; 1.8)

Some data were previously published in other studies within the main research project [12,53,54]. *CMKLR1*, Chemerin receptor 1 gene; s-CRP, serum C-reactive protein; *GAPDH*, glyceraldehyde 3-phosphate dehydrogenase gene; s-Endocan, serum endocan; s-E-selectin, serum E-selectin; s-IL-6, serum interleukin 6; s-IL-1β, serum interleukin 1 beta; s-IL-10, serum interleukin 10; SPMs, specialized proresolving mediators; s-RvD1, serum resolvin D1; s-RvE1, serum resolvin E1; s-TNF-α, serum tumor necrosis factor alpha; s-VCAM-1, serum vascular cell adhesion molecule 1; VV-ECMO, veno-venous extracorporeal membrane oxygenation. Results are expressed as mean ± SEM or as median (25th percentile; 75th percentile) for data with normal or non-normal distribution, respectively. RvE1 receptor results are expressed as arbitrary units. * *p* < 0.050 vs. Admission; ** *p* < 0.010 vs. Admission; *** *p* < 0.001 vs. Admission. Statistically significant differences compared to admission are highlighted in bold.

**Table 3:** Repeated measures multivariate models for s-ANXA1 in all COVID-19 patients.

	Adjusted β	95% CI	*p* Value
**s-ANXA1 (ng/mL) (Admission to Week 2)**			
Model 1			
COVID-19 Groups			
Severe	Ref		
Critical	2.42	−4.38 to 9.22	0.486
Critical on VV-ECMO	3.31	−2.91 to 9.53	0.296
Age (Years)	0.08	−0.07 to 0.24	0.291
Sex			
Male	Ref		
Female	−4.28	−9.17 to 0.61	0.086
Model 2			
s-RvE1 (pg/mL) (admission to week 2)	**0.019**	**0.012 to 0.026**	**<0.001**
Age (Years)	0.09	−0.05 to 0.22	0.210
Sex			
Male	Ref		
Female	−1.72	−6.91 to 3.47	0.516
Model 3			
Intrahospital mortality			
Non-survivors	Ref		
Survivors	−1.87	−9.33 to 5.60	0.624
Age (Years)	0.04	−0.10 to 0.18	0.573
Sex			
Male	Ref		
Female	**−5.29**	**−10.24 to** **−0.34**	**0.036**
**s-ANXA1 (ng/mL) (Week 3 and 4)**			
Model 4			
Intrahospital mortality			
Non-survivors	Ref		
Survivors	8.48	−2.54 to 19.49	0.131
Age (Years)	0.18	−0.21 to 0.57	0.369
Sex			
Male	Ref		
Female	**−9.97**	**−17.60 to** **−2.34**	**0.010**

Adjusted β, 95% confidence intervals (95% CI) and *p* value estimated by repeated measures multivariate models with s-ANXA1 as the dependent variable and adjusted for age and sex. Significant associations are highlighted in bold. Ref, reference; s-ANXA1, serum annexin A1; s-RvE1, serum resolvin E1; VV-ECMO, veno-venous extracorporeal membrane oxygenation.

## Data Availability

The datasets used and/or analyzed during the current study are available from the corresponding authors on reasonable request due to privacy.

## Supplementary Materials

The following supporting information can be downloaded at: https://www.mdpi.com/article/10.3390/biom16040508/s1, Table S1: Final number per group for each of the parameters at admission; Table S2: Correlations for s-ANXA1 and *FPR2* in all COVID-19 patients during hospitalization; Figure S1: Flowchart showing the patients’ groups classification and indicating the number of severe COVID-19, critical COVID-19 and critical COVID-19 on VV-ECMO patients analyzed at each time point and the reasons for missing data and patient drop out from the study; Figure S2: Number of COVID-19 patients exposed to a cumulative dose of 6–12 mg/kg, 18–30 mg/kg and 36–60 mg/kg of dexamethasone at each time point of sample collection, considering the treatment regimen of 6 mg/kg/day. Additionally, the number of COVID-19 patients that completed the treatment (off dex) and were under or off dexamethasone effect, according to its biological half-life of 36–54 h, is presented; Figure S3: s-ANXA1 in dexamethasone-treated COVID-19 patients categorized by the cumulative dose of dexamethasone at each time point, and after treatment completion (off dex), whether under or off dexamethasone effect, according to its biological half-life of 36–54 h; Figure S4: *FPR2* mRNA expression in dexamethasone-treated COVID-19 patients categorized by the cumulative dose of dexamethasone at each time point, and after treatment completion (off dex), whether under or off dexamethasone effect, according to its biological half-life of 36–54 h.
