# Supplementary material for: Annexin A1 and Dexamethasone Treatment in Hospitalized COVID-19 Patients: Impact on Disease Recovery and Evidence for an Interplay Between Proresolving Mediators
Source: Biomolecules. 2026 Mar 28;16(4):508. doi: 10.3390/biom16040508 (PMC13113340; doi:10.3390/biom16040508)
Supplement: Supplementary file 1 [file biomolecules-16-00508-s001.zip › biomolecules-4160898-supplementary.pdf]

**Table S1** Final number per group for each of the parameters at admission.

| **Biomarkers/Parameters** | **Controls** (n) | **Severe COVID-19** (n) | **Critical COVID-19** (n) | **Critical COVID-19 on VV-ECMO** (n) |
|---|---|---|---|---|
| **APACHE II score** | - | - | 17 | 17 |
| **SAPS II score** | - | - | 17 | 17 |
| **$PaO_2$/$FiO_2$ ratio** | - | 25 | 17 | 11 |
| **$PaCO_2$** (mmHg) | - | 25 | 17 | 17 |
| **Lactate** (mmol/L) | - | 21 | 15 | 16 |
| ***Inflammatory Parameters*** | | | | |
| *Cytokines* | | | | |
| **s-TNF-α** (pg/mL) | 23 | 27 | 17 | 17 |
| **s-IL-1β** (pg/mL) | 23 | 27 | 17 | 17 |
| **s-IL-6** (pg/mL) | 22 | 27 | 17 | 17 |
| **s-IL-10** (pg/mL) | 23 | 27 | 17 | 17 |
| *Inflammatory marker* | | | | |
| **s-CRP (mg/L)** | - | 27 | 17 | 17 |
| *Inflammatory cells* | | | | |
| **Leukocytes** (× $10^9$/L) | - | 27 | 17 | 16 |
| **Neutrophils** (× $10^9$/L) | - | 27 | 17 | 15 |
| **Monocytes** (× $10^9$/L) | - | 27 | 17 | 15 |
| **Lymphocytes** (× $10^9$/L) | - | 27 | 17 | 15 |
| ***Proresolving Parameters*** | | | | |
| **s-ANXA1** (ng/mL) | 23 | 27 | 17 | 17 |
| **s-RvD1** (pg/mL) | 23 | 27 | 17 | 17 |
| **s-RvE1** (pg/mL) | 23 | 27 | 17 | 17 |
| **FPR2** (mRNA expression) | 21 | 18 | 15 | 13 |
| **$Chemerin_1$** (mRNA expression) | 19 | 18 | 15 | 13 |
| ***Endothelial Activation Markers*** | | | | |
| **s-Endocan** (ng/mL) | 23 | 27 | 17 | 17 |
| **s-VCAM-1** (ng/mL) | 23 | 27 | 17 | 17 |
| **s-E-selectin** (ng/mL) | 23 | 27 | 17 | 17 |

APACHE II, acute physiology and chronic health evaluation II; $Chemerin_1$, chemerin receptor 1; $FiO_2$, fraction of inspired oxygen; s-ANXA1, serum annexin A1; s-Endocan, serum endocan; s-E-selectin, serum E-selectin; s-IL-1β, serum interleukin 1 beta; s-IL-6, serum interleukin 6; s-IL-10, serum interleukin 10; s-RvD1, serum resolvin D1; s-RvE1, serum resolvin E1; s-TNF-α, serum tumor necrosis factor alpha; s-VCAM-1, serum vascular cell adhesion molecule 1; SAPS II, Simplified Acute Physiology Score II; $PaCO_2$, partial pressure of arterial carbon dioxide; $PaO_2$, Partial pressure of arterial oxygen; VV-ECMO, veno-venous extracorporeal membrane oxygenation.

**Table S2.** Correlations for s-ANXA1 and FPR2 in all COVID-19 patients during hospitalization.

| | s-ANXA1 (ng/mL) | | | | | | | | | |
|---|---|---|---|---|---|---|---|---|---|---|
| | Admission | | Days 3-4 | | Days 5-8 | | Week 2 | | Week 3 | |
| | r Spearman | Adjusted *P* | r Spearman | Adjusted *P* | r Spearman | Adjusted *P* | r Spearman | Adjusted *P* | r Spearman | Adjusted *P* |
| **s-RvE1 (pg/mL)** | **0.562** | **<0.005** | **0.416** | **0.019** | **0.418** | **0.028** | 0.447 | 0.073 | 0.175 | 0.721 |
| ***CMKLR1/ GAPDH*** **(mRNA expression)** | **−0.503** | **0.010** | −0.209 | 0.386 | −0.113 | 0.720 | −0.135 | 0.801 | 0.006 | >0.999 |
| **s-IL-1β (pg/mL)** | 0.244 | 0.161 | 0.277 | 0.150 | 0.034 | 0.959 | −0.034 | 0.974 | −0.271 | 0.451 |
| **Leukocytes (× $10^9$/L)** | **0.493** | **<0.005** | 0.295 | 0.153 | **0.452** | **0.020** | 0.313 | 0.258 | 0.318 | 0.380 |
| **Neutrophils (× $10^9$/L)** | **0.442** | **0.008** | 0.281 | 0.157 | **0.495** | **0.011** | 0.157 | 0.724 | 0.304 | 0.439 |
| **Monocytes (× $10^9$/L)** | 0.286 | 0.128 | 0.055 | 0.909 | 0.044 | 0.933 | 0.105 | 0.791 | 0.349 | 0.390 |
| **Lymphocytes (× $10^9$/L)** | 0.266 | 0.148 | 0.142 | 0.581 | 0.116 | 0.683 | 0.224 | 0.484 | 0.185 | 0.701 |
| **s-Endocan (ng/mL)** | 0.073 | 0.804 | −0.288 | 0.152 | −0.050 | 0.901 | −0.473 | 0.052 | −0.084 | 0.895 |
| **s-VCAM-1 (ng/mL)** | −0.286 | 0.129 | −0.186 | 0.399 | 0.026 | 0.960 | 0.183 | 0.594 | 0.032 | 0.946 |
| **s-E-Selectin (ng/mL)** | −0.005 | >0.999 | −0.023 | 0.943 | 0.205 | 0.389 | −0.143 | 0.704 | −0.436 | 0.150 |
| | **FPR2 mRNA expression** | | | | | | | | | |
| | **Admission** | | **Days 3-4** | | **Days 5-8** | | **Week 2** | | **Week 3** | |
| | r Spearman | Adjusted *P* | r Spearman | Adjusted *P* | r Spearman | Adjusted *P* | r Spearman | Adjusted *P* | r Spearman | Adjusted *P* |
| **s-RvE1 (pg/mL)** | 0.350 | 0.128 | 0.151 | 0.568 | −0.089 | 0.697 | 0.253 | 0.584 | 0.200 | 0.730 |
| **s-TNF-α (pg/mL)** | 0.331 | 0.124 | −0.233 | 0.294 | −0.134 | 0.584 | −0.040 | 0.874 | 0.091 | 0.869 |
| **s-IL-6 (pg/mL)** | **0.641** | **<0.002** | 0.285 | 0.267 | 0.266 | 0.335 | 0.375 | 0.314 | 0.297 | 0.610 |

*P* values were adjusted for multiple comparisons using the Benjamini-Hochberg false discovery rate correction. Bold values are shown for parameters with statistically significant correlations. *CMKRL1*, Chemerin1 gene; *GAPDH*, glyceraldehyde 3-phosphate dehydrogen-ase gene; s-ANXA1, serum annexin A1; s-Endocan, serum endocan; s-E-Selectin, serum E-Selectin; s-IL-6, serum interleukin 6; s-IL-1β, serum interleukin 1 beta; s-RvE1, serum resolvin E1; s-TNF-*α*, serum tumor necrosis factor alpha; s-VCAM-1, serum vascular cell adhesion molecule 1.

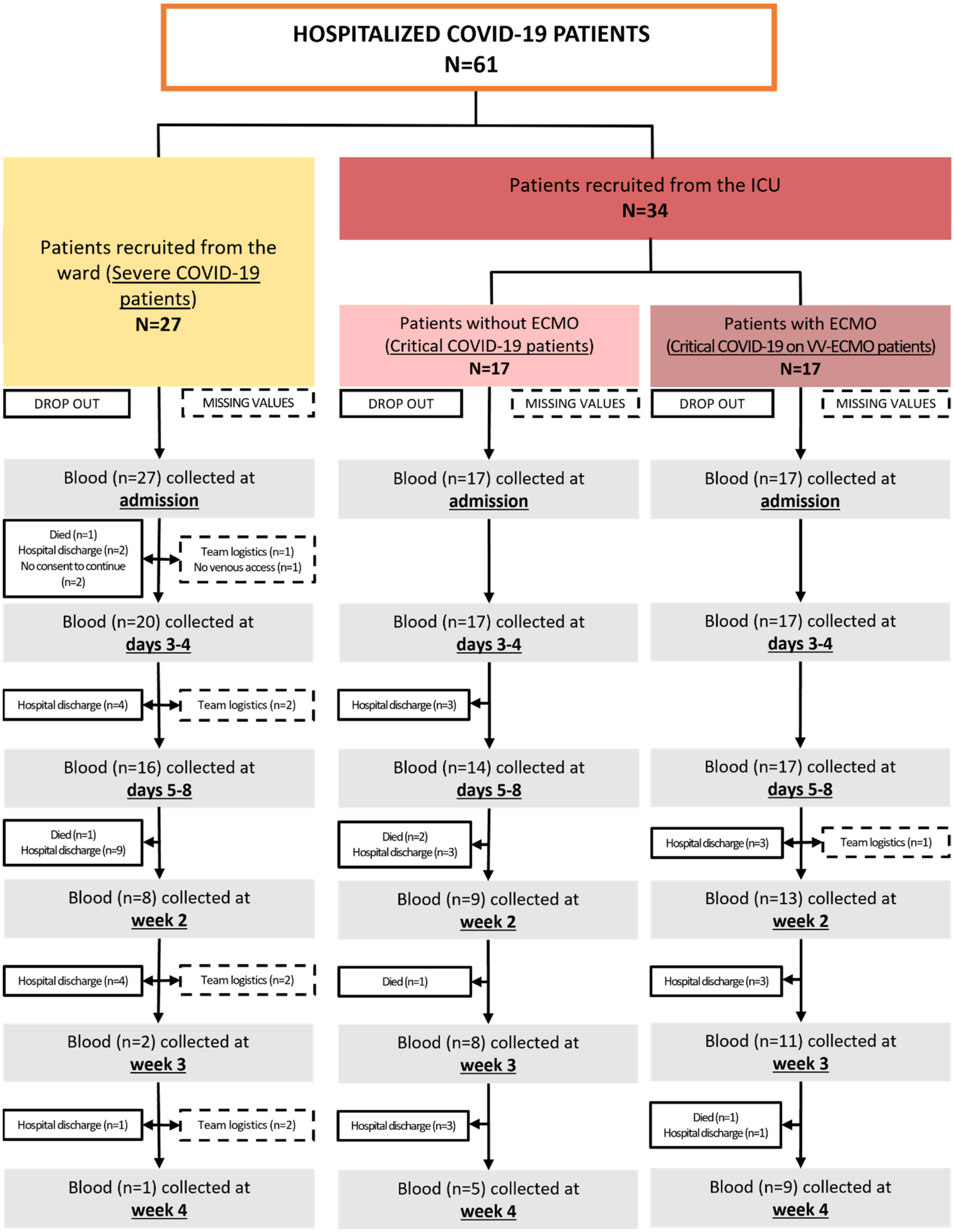


**Figure S1.** Flowchart showing the patients' groups classification and indicating the number of severe COVID-19, critical COVID-19 and critical COVID-19 on VV-ECMO patients analysed at each time point and the reasons for missing data and patient drop out from the study. ECMO, extracorporeal membrane oxygenation; ICU, intensive care unit; VV-ECMO, veno-venous extracorporeal membrane oxygenation.

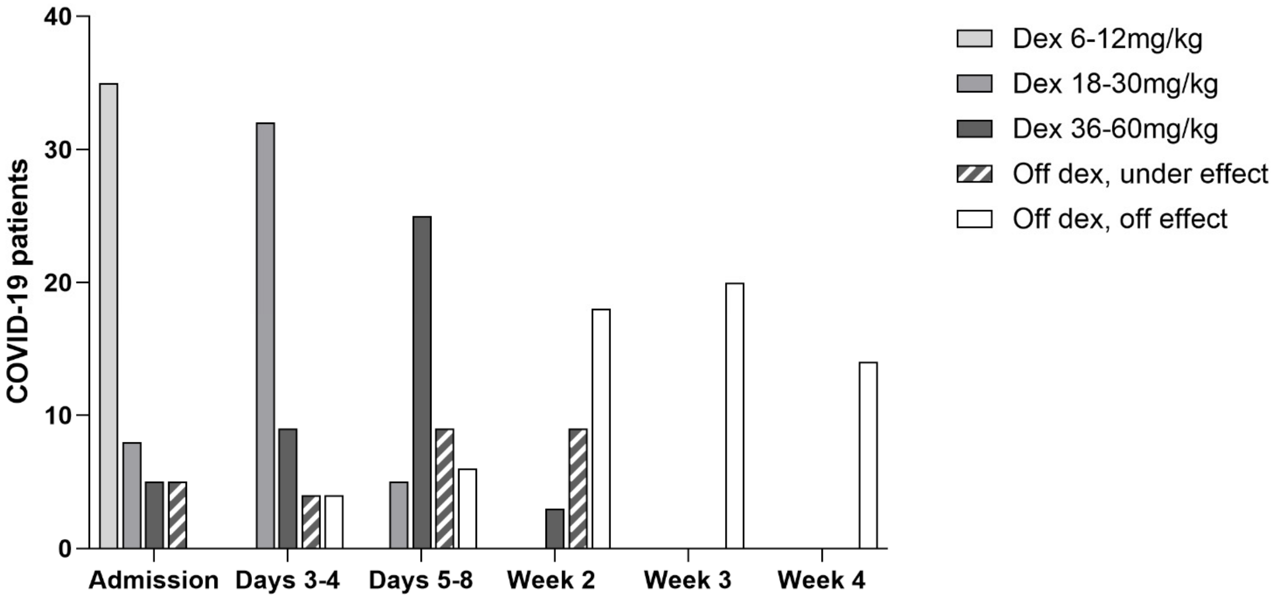


**Figure S2.** Number of COVID-19 patients exposed to a cumulative dose of 6-12mg/kg, 18-30mg/kg and 36-60mg/kg of dexamethasone at each time point of sample collection, considering the treatment regimen of 6mg/kg/day. Additionally, the number of COVID-19 patients that completed the treatment (off dex) and were under or off dexamethasone effect, according to its biological half-life of 36-54 hours, is presented. Dex, dexamethasone.

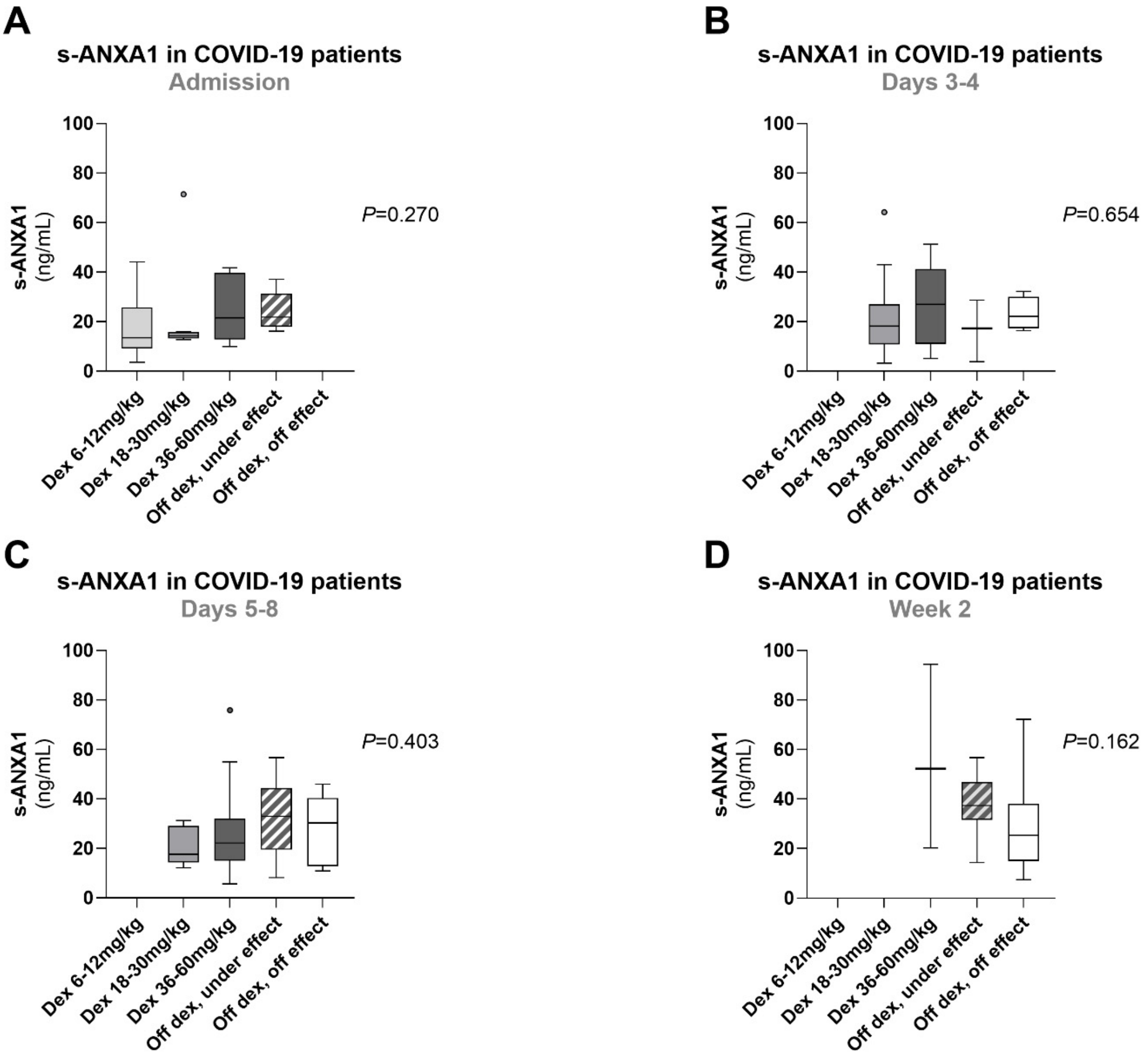


**Figure S3.** s-ANXA1 in dexamethasone-treated COVID-19 patients categorized by the cumulative dose of dexamethasone at each time point, and after treatment completion (off dex), whether under or off dexamethasone effect, according to its biological half-life of 36-54 hours. Dex, dexamethasone.

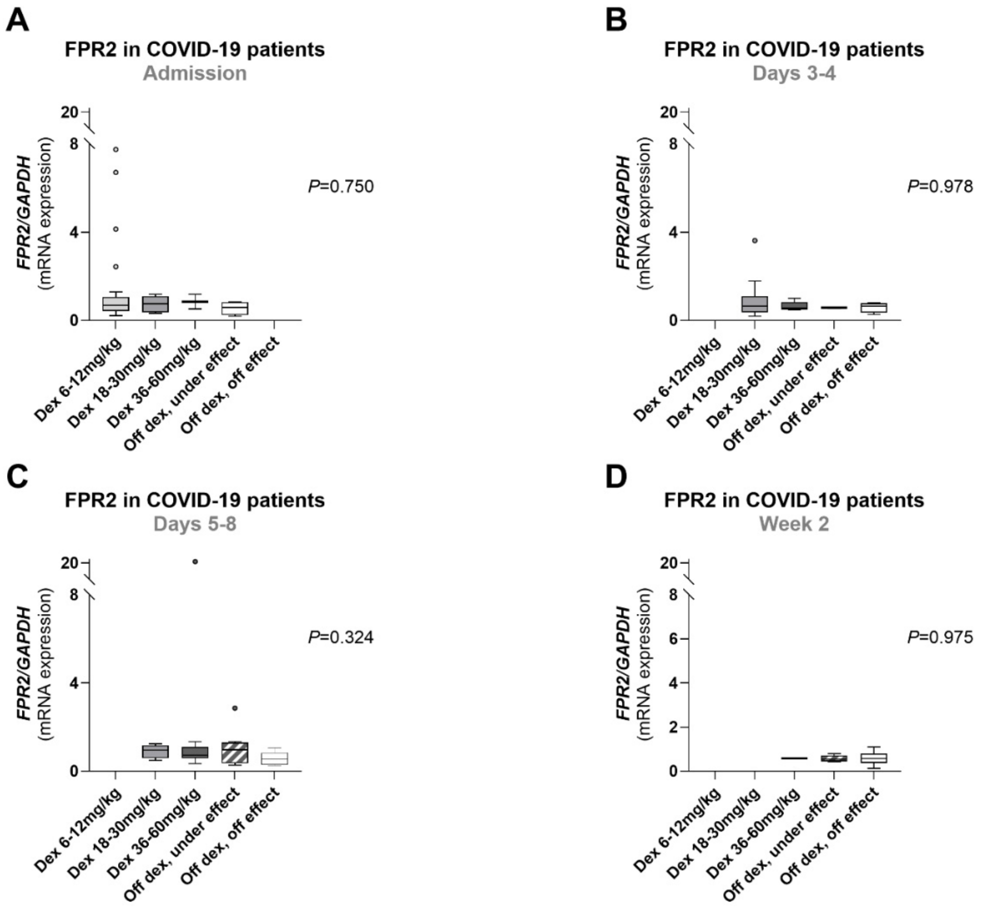


**Figure S4.** FPR2 mRNA expression in dexamethasone-treated COVID-19 patients categorized by the cumulative dose of dexamethasone at each time point, and after treatment completion (off dex), whether under or off dexamethasone effect, according to its biological half-life of 36-54 hours. Dex, dexamethasone.
